# Elucidation of the interplay between Fe(II), Fe(III), and dopamine with relevance to iron solubilization and reactive oxygen species generation by catecholamines

**DOI:** 10.1111/jnc.13615

**Published:** 2016-05-12

**Authors:** Yingying Sun, A. Ninh Pham, T. David Waite

**Affiliations:** ^1^ School of Civil and Environmental Engineering The University of New South Wales Sydney New South Wales Australia

**Keywords:** dopamine, H_2_O_2_, iron, kinetics, neurodegenerative disorders, oxidation

## Abstract

The non‐enzymatically catalyzed oxidation of dopamine (DA) and the resultant formation of powerful oxidants such as the hydroxyl radical (^•^ OH) through ‘Fenton chemistry’ in the presence of iron within dopaminergic neurons are thought to contribute to the damage of cells or even lead to neuronal degenerative diseases such as Parkinson's disease. An understanding of DA oxidation as well as the transformation of the intermediates that are formed in the presence of iron under physiological conditions is critical to understanding the mechanism of DA and iron induced oxidative stress. In this study, the generation of H_2_O_2_ through the autoxidation and iron‐catalyzed oxidation of DA, the formation of the dominant complex via the direct reaction with Fe(II) and Fe(III) in both oxygen saturated and deoxygenated conditions and the oxidation of Fe(II) in the presence of DA at physiological pH 7.4 were investigated. The oxidation of DA resulted in the generation of significant amounts of H_2_O_2_ with this process accelerated significantly in the presence of Fe(II) and Fe(III). At high DA:Fe(II) ratios, the results from this study suggest that DA plays a protective role by complexing Fe(II) and preventing it from reacting with the generated H_2_O_2_. However, the accumulation of H_2_O_2_ may result in cellular damage as high intracellular H_2_O_2_ concentrations will result in the oxidation of remaining Fe(II) mainly through the peroxidation pathway. At low DA:Fe(II) ratios however, it is likely that DA will act as a pro‐oxidant by generating H_2_O_2_ which, in the presence of Fe(II), will result in the production of strongly oxidizing ^•^ OH radicals.

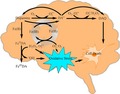

Powerful oxidants such as the hydroxyl radical (^•^OH) have previously been thought to be generated through the interplay between dopamine (DA) and iron, contributing to damage to cells and, potentially, leading to neuronal degenerative diseases such as Parkinson's disease. Our results suggest that DA plays a dual role as high DA/Fe(II) ratios prevent Fe(II) from reacting with the generated H_2_O_2_ thereby reducing ^•^OH generation, whereas low DA/Fe(II) ratios enhance ^•^OH generation as a result of reaction of unbound Fe(II) and H_2_O_2_ produced via both autoxidation and iron‐catalyzed oxidation of DA.

**Abbreviations used**
^•^OH: hydroxyl radicals
DA^•‐^: semiquinone radical
$O_{2}^{∙-}$: superoxide
6‐OHDA: 6‐hydroxydopamine
AD: Alzheimer's disease
ADHD: attention deficit hyperactivity disorder
AFO: amorphous iron oxide
DAC: dopaminochrome
DA: dopamine
DAL: leukoaminochrome
DAQ: dopamine‐*o*‐quinone
DHI: 5,6‐dihydroxyindole
DPD: *N*,*N*‐diethyl‐*p*‐phenylenediamine
DTPA: diethylenetriaminepentaacetic acid
Fe(II): inorganic ferrous ion
Fe(III): inorganic ferric ion
Fe(III)_I_: total inorganic Fe(III)
FZ: 3‐(2‐pyridyl)‐5,6‐diphenyl‐1,2,4‐triazine‐*p,p′*‐disulfonic acid
H_2_O_2_: peroxide
HRP: horseradish peroxidase
MOPS: 3‐(N‐morpholino) propanesulfonic acid
MQ: Milli‐Q water
PD: Parkinson's disease
RLS: restless leg syndrome
ROS: reactive oxygen species
SN: substantia nigra
VBA: Visual Basic for Applications

Dopamine (1‐amino‐2‐(3,4‐dihydroxyphenyl)ethane, DA) is a well‐known neurotransmitter in humans (Hornykiewicz 1966). In the human brain, DA is mainly stored in the caudate, putamen, and substantia nigra at micromolar to millimolar concentrations (Hardy *et al*. 1987; Staal *et al*. 2004). In the presence of oxygen, the generation of the dopamine semiquinone radical (DA^•−^) and dopamine‐*o*‐quinone (DAQ) from the non‐enzymatic autoxidation of DA results in the production of reactive oxygen species (ROS), including peroxide (H_2_O_2_) and superoxide ($O_{2}^{∙-}$) (Graham *et al*. 1978), which may further participate in metal‐catalyzed reactions to form much more strongly oxidizing ROS, such as hydroxyl radicals (^•^OH). It is reported that both the dopamine‐derived quinones and concomitant generation of ROS are associated with neurodegenerative disorders or even lead to cancer and cell death as a result of their ability to cause damage to lipids, proteins, and DNA (Taketani 2005; Zafar *et al*. 2006; Vashchenko and MacGillivray 2013). Thus, better understanding of the transformation of DA and generation of ROS under physiological conditions, especially in the presence of metals would help elucidate the causes of these various neurodegenerative disorders and develop soundly based approaches to prevention and treatment.

Iron, an indispensable nutrient and a cofactor for many proteins (Greene *et al*. 1994), plays a crucial role for almost all types of cells in the human body (Batista‐Nascimento *et al*. 2012). As the most abundant transition metal within the brain, the homeostasis of iron is essential to maintaining a healthy brain capable of affecting the synthesis and signaling of neurotransmitters such as DA (Meiser *et al*. 2013), ATP generation (Lill and Mühlenhoff 2006) and myelination (Ortiz *et al*. 2004). However, intracellular iron levels must be tightly regulated as a deficiency in this critical element is commonly associated with dysfunction in movement (Kastman *et al*. 2010), mental problems (Hurtado *et al*. 1999) and limitations in iron‐dependent antioxidant production (Wan *et al*. 2012) and has been reported to lead to diseases such as attention deficit hyperactivity disorder (Cortese *et al*. 2012) and restless leg syndrome (Connor *et al*. 2011; Allen *et al*. 2013). Conversely, excessive concentrations of iron may lead to cell damage as a result of so‐called ‘Fenton chemistry’ (Vashchenko and MacGillivray 2013) and is widely associated with neurodegenerative diseases such as Alzheimer's disease (Antharam *et al*. 2012) and Parkinson's disease (PD) (Kosta *et al*. 2006; Peng *et al*. 2010; Chew *et al*. 2011). In the healthy brain, excess iron is generally sequestered by ferritin, a primary iron‐storage protein capable of accommodating up to 4500 iron atoms, and stored in the relatively inert ferrihydrite core until it is needed for critical iron‐dependent metabolic processes (Reif 1992; Harrison and Arosio 1996).

It has previously been shown that the oxidation of DA can be catalyzed in the presence of iron (Chinta and Andersen 2008; Jiang *et al*. 2013) resulting in an abnormal accumulation of the intermediate oxidation product 6‐hydroxydopamine (Pezzella *et al*. 1997) and enhanced formation of neuromelanin, the end‐product of the oxidation of dopamine (Sulzer *et al*. 2000). The presence of this polymeric material in the brain has been proposed to both induce and alleviate PD (Double *et al*. 2002; Segura‐Aguilar *et al*. 2014) with the ability of iron to catalyze its formation suggesting a role of iron in the pathogenicity of PD (Martin *et al*. 2008; Berg *et al*. 2011). In addition, DA can form a variety of complexes with both Fe(II) and Fe(III) over a range of pH (Avdeef *et al*. 1978; Crisponi *et al*. 1983) with the association of DA with iron certain to exert a significant impact on both Fe(II) and Fe(III) redox transformations and the associated generation of ROS. It has previously been proposed that dopamine and its reductive intermediates, DA^•−^, and 6‐hydroxydopamine can mobilize ferritin‐bound iron (Montiero *et al*. 1989; Reif 1992; Double *et al*. 1998) through a reduction process [in which Fe(III) is reduced to the highly soluble Fe(II)] possibly by initially forming a complex with Fe(III) on the surface of the ferritin (Sánchez *et al*. 2005) with the free ferrous ion resulting from reduction in the Fe(III) metal center resulting in the generation of ^•^OH through ‘Fenton chemistry’.

While the ability of catechol‐like compounds such as dopamine to enhance the oxidation of Fe(II) and to form complexes with both Fe(II) and Fe(III) (Powell and Taylor 1982; Naka *et al*. 2006) has been extensively investigated (Perron *et al*. 2010; García *et al*. 2012), most of the previous studies have focused on the cell damage arising from the generation of ROS, especially through ‘Fenton chemistry’ (Hermida‐Ameijeiras *et al*. 2004) rather than the ability of these redox‐active compounds to mediate the electron transfer between iron and themselves. Moreover, even though the formation of Fe(III)‐DA complexes through the direct reaction of DA and iron and the subsequent reduction in the Fe(III) metal‐center and release of Fe(II) have long been recognized to occur (Avdeef *et al*. 1978; El‐Avaan *et al*. 1997; Hermida‐Ameijeiras *et al*. 2004), very little quantification of the rate constants associated with these dynamic processes has been undertaken at physiological pH with the result that our ability to predict the impact of increase or decrease in iron levels in the brain on iron‐induced oxidative stress is very limited. In addition, a number of the previous studies have used extremely high iron concentrations (Jiang *et al*. 2013), low pH (García *et al*. 2012) or complexing buffer solutions (Napolitano *et al*. 1999) which may well have resulted in complications in interpretation of the results obtained. Given the widespread distribution of iron, DA, and its analogous compounds in the human brain, it is imperative to understand the mechanisms that govern their cytotoxicity at physiological pH.

In this study, the kinetics and mechanism of the interaction of DA with Fe(II) and Fe(III) are investigated at physiological pH of 7.4 over a range of metal to ligand ratios under both deoxygenated and oxygen‐saturated conditions, with emphasis on the generation of H_2_O_2_ through both DA autoxidation and iron‐catalyzed DA oxidation, formation of Fe(III)‐DA complexes and the oxidation of Fe(II) through the direct DA‐iron reaction. Based on the experimental data, a detailed kinetic model capable of describing the complicated interplay between both Fe(II) and Fe(III) species, the iron oxide ferrihydrite, DA, oxygen, and the intermediate oxidation products is developed.

## Materials and methods

All analytical grade chemicals were purchased from Sigma‐Aldrich (Castle Hill, NSW, Australia) (or as otherwise stated) and were used without further refinement. All solutions were prepared using 18 MΩ cm ultrapure Milli‐Q water (MQ) (Merck Millipore Corporation, Darmstadt, Germany). All glassware was acid washed in 5% v/v HCl for at least 1 week before use. Stock solutions were kept in dark bottles and were refrigerated at 4°C when not in use. All experiments were conducted under dark conditions and performed at a controlled room temperature of 22 ± 0.6°C.

Solutions were prepared at pH 7.4 by adding an appropriate amount of concentrated NaOH and HCl to buffer solutions containing 0.1 M NaCl, 2 mM NaHCO_3_, and 10 mM 3‐(N‐morpholino) propanesulfonic acid (MOPS). Low concentrations of MOPS were found to have a negligible effect on the rates of Fe(II) oxidation (Sun *et al*. 2015). All pH measurements were conducted using a Hanna HI9025 pH meter combined with a glass electrode and Ag/AgCl reference. Calibration of the pH electrode was undertaken using NIST buffer solutions (pH 7.01 and 10.01). Experiments were conducted in darkness with the reactor covered in foil for the duration of the reaction.

A concentrated Fe(II) stock solution (5 mM) was prepared by dissolving ferrous ammonium sulfate hexahydrate (Fe(NH_4_SO_4_)_2_?6H_2_O) in 5 mM HCl. Concentrated stock solutions of 10 mM Fe(III) [using ferric chloride hexahydrate (FeCl_3_·6H_2_O)] and 10 mM DA were prepared weekly in 10 mM HCl. The working stock solutions of Fe(II), Fe(III), and DA were diluted from the concentrated stock solutions daily in 10 mM HCl solution. The acidity of both concentrated stock and working stock solutions was sufficient to avoid significant oxidation of Fe(II) and DA and precipitation of Fe(III) on the time scale of interest and yet low enough to minimize any pH change that might occur on addition of the stock to experimental solutions. A stock solution of 20 mM H_2_O_2_ prepared by dilution of a nominal 30% w⁄w H_2_O_2_ solution was used for calibration of the H_2_O_2_ measurements. The nominal 30% w⁄w solution was standardized by UV spectrophotometry at 240 nm (Morgan *et al*. 1988). A ferrozine (FZ) working solution of 50 mM was prepared by dissolving the hydrated monosodium salt of 3‐(2‐pyridyl)‐5,6‐diphenyl‐1,2,4‐triazine‐*p,p′*‐disulfonic acid in MQ water. Stock solutions of 60 mM *N*,*N*‐diethyl‐*p*‐phenylenediamine (DPD) and 50 KU/L horseradish peroxidase (HRP) were prepared in MQ water as described in Bader *et al*. (1988). A 10 mM stock solution of diethylenetriaminepentaacetic acid (DTPA) was prepared in MQ water and the pH adjusted to 8.0 by adding concentrated NaOH. A 3000 KU/L stock solution of superoxide dismutase (SOD from bovine erythrocytes containing Cu and Zn) was prepared in MQ and stored at −85°C prior to use. Stock solutions of 10 mM 1,10‐phenanthroline were prepared in 5 mM HCl.

### Measurement of Fe(II) concentration

The concentration of Fe(II) was quantified spectrophotometrically using the FZ method (Stookey 1970; Viollier *et al*. 2000) in a 10 cm cuvette by a Cary 60 spectrophotometer at 562 nm and baseline corrected at 690 nm. FZ was chosen because it reacts extremely rapidly with Fe(II) to form a stable purple complex (Fe^II^FZ_3_) with a maximum absorbance at 562 nm and molar absorptivity of ε_562 nm_ = 30 000/M/cm (Stookey 1970; Viollier *et al*. 2000). The influence of Fe^III^DA_2_ complexes on the measurement of Fe^II^FZ_3_ was eliminated by subtracting its baseline corrected absorbance at 562 nm.

### Measurement of H_2_O_2_ concentration

The H_2_O_2_ formed during the course of oxidation of DA [both in the absence and presence of Fe(II) or Fe(III)] was quantified using the photometric DPD method (Bader *et al*. 1988). Briefly, DPD is oxidized by H_2_O_2_ with a 2 : 1 stoichiometry in the presence of HRP, resulting in an absorbance at both 551 nm and 510 nm with molar extinction coefficients at these wavelengths of 21 000 ± 500/M/cm and 19 800 ± 500/M/cm, respectively. The detection limit of this method is ~ 10 nM in aqueous solutions when 10 cm cuvettes are used. To halt the continuous generation of H_2_O_2_ during the measurement, 1 mM DTPA was added to bind any iron present in the solution. At this concentration, DTPA can compete effectively with DA for both the added or generated Fe(III) and Fe(II) given the relative strengths of the DA and DTPA complexes with Fe(II) [log*K*_Fe(II)+DA_ = 7.95 << log*K*_Fe(II)+DTPA_ = 16.4 (Martell and Smith 1974; Smith and Martell 1989)] and Fe(III) [log*K*_Fe(III)+DA_ = 20.0 << log*K*_Fe(III)+DTPA_ = 28 (Martell and Smith 1974; Avdeef *et al*. 1978)]. To eliminate the influence of Fe(II) on the measurement of H_2_O_2_, 500 μM 1,10‐phenanthroline was added before the addition of DTPA for the high Fe(II)/DA ratio condition. The system was calibrated by adding standard H_2_O_2_ stock into the buffer solutions, along with a zero standard containing 60 μM DPD and 500 U/L HRP. Generally, two sets of calibration [0–100 nM (in the presence of phenanthroline) and 0–1500 nM (in the absence of phenanthroline)] were undertaken with calibration curves constructed by linear regression of the calibration data. Interference arising from the presence of low concentration of DA was found to be negligible for the measurement of H_2_O_2_ in the presence of phenanthroline (data measured is not provided here). Interference arising from the presence of Fe(III) and DA was found to be negligible under the experimental conditions investigated here (Figure S1).

### Measurement of Fe(III)‐DA complexes

Similar to a range of previously reported catecholate compounds [including catechol, tiron, 2,3‐dihydroxybenzoic acid and 3,4‐dihydroxyphenylalanine (DOPA)], addition of Fe(III) into solutions containing DA is expected to produce three different complexes: the *mono*‐complex (denoted hereafter as Fe^III^DA), the *bis*‐complex (denoted hereafter as Fe^III^DA_2_), and the *tris*‐complex (denoted hereafter as Fe^III^DA_3_) with the dominant species being pH and concentration‐dependent (Avdeef *et al*. 1978; Sever and Wilker 2004). The molar absorptivities for the three analogous Fe(III)‐catecholate complexes are ε_714 nm_ = 1000/M/cm, ε_570 nm_ = 3330/M/cm and ε_490 nm_ = 4190/M/cm, respectively (Sever and Wilker 2004). Thermodynamic modeling of the effect of DA concentration and pH on the proportions of the various Fe(III)‐DA complexes present (Figures S2a, b and c) reveals that the *bis*‐complex Fe^III^DA_2_ is expected to be the dominant species present under the conditions investigated in this study.

The concentration of Fe^III^DA_2_ was determined spectrophotometrically by measuring the absorbance at 570 nm using a Cary 60 spectrophotometer with baseline correction at 850 nm (Gerard *et al*. 1994; Charkoudian and Franz 2006). A calibration curve for quantification of the concentration of the Fe^III^DA_2_ complex (Figure S3) was developed under deoxygenated conditions to prevent any oxidation and transformation of the complex. Briefly, the solutions were sparged for 1 h using a special gas mixture of 297 ± 6 ppm CO_2_ in argon (BOC) prior to the addition of DA. The solution was then bubbled for another 10 min before the addition of Fe(III). Continuous sparging was maintained during the course of the deoxygenated experiment. The molar absorptivity of Fe^III^DA_2_ was calculated to be 3312/M/s which is quite similar to that of the *bis*‐complex Fe(III)‐catechol. It should be noted that although the *tris*‐complex Fe^III^DA_3_ has a higher absorptivity than that of the *bis‐*Fe^III^DA_2_ (data measured under deoxygenated conditions is not given here), it would be expected to have minimal effect on the measurement of Fe^III^DA_2_ in view of its very low concentrations under the experimental conditions investigated here. In addition, Fe(II)‐DA complexes are colorless (data measured under deoxygenated conditions is not provided here) (Tyson and Martell 1968; Powell and Taylor 1982) and as such would not affect the measurement of Fe^III^DA_2_.

### Speciation modeling

Knowledge of the speciation of both Fe(II) and Fe(III) in the presence of various concentrations of DA was utilized to determine the major iron‐DA complexes present at particular concentrations of added iron and DA. Fe(II) and Fe(III) speciation calculations were undertaken using the program Visual Minteq (Gustafsson 2005) with the equilibrium reactions and stability constants used provided in Table S1.

### Kinetic modeling

The autoxidation of DA and its interaction with Fe(II) or Fe(III) were modeled using a simplistic approach originally developed by Rose and Waite (2002). In this model, ‘Fe(II)’ and ‘Fe(III)’ represent all inorganically complexed species of ferrous and ferric ions, respectively (details of the species are shown in Table S1). The relative proportions of these species vary with pH and DA concentration (Avdeef *et al*. 1978; Hider *et al*. 1981; King *et al*. 1995).

Considering the likely complexity of the mechanism, the kinetic model was developed progressively by using the kinetic modeling program Kintek Explorer to fit the experimental data over a range of conditions (Johnson *et al*. 2009). Briefly, the autoxidation of DA in the absence of iron was firstly fitted followed by fitting of the interaction between Fe(III) and DA in both deoxygenated and oxygenated conditions by considering the generation of H_2_O_2_ and the formation of Fe^III^DA_2_ (as shown in Figs. S2a, b and c), the major Fe(III)‐DA complex under the conditions of interest. The interplay of Fe(II) and DA in the presence of O_2_ was finally fitted because of the complicated transformation between Fe(II) and Fe(III) with the oxidation of Fe(II) also accounted for in the model fitting. In the proposed reaction scheme (Tables 1, 2, 3), rate constants for reactions 1, 6, 8, 10, 11, 12, 15, 17, 18, 19, 22, 23, and 26 are the model fitting parameters at various ligand to metal ratios. Rate constants of other reactions were obtained from the literature (as described in the Tables 1, 2, 3 footnotes). The sensitivity of the model to changes in individual rate constant values, defined as the relative residual, *r*, was assessed by examining the change in the relative difference between the experimental data and the kinetic model simulation when one rate constant was varied with the others fixed at their optimal values. The program Kintecus (Ianni 2012) combined with a Visual Basic for Applications program was used to calculate *r* with results of this sensitivity analysis shown in Fig. S4.

**Table 1:** Modeled reactions and rate constants for the autoxidation of DA

No.	Reactions	Rate constants (per Ms or per s)	Reference
1	$\text{DA}+O_{2}\overset{k_{1}}{⟶}O_{2}^{∙-}+{\text{DA}}^{∙-}$	*k*_1_ = 9.29 × 10^−3^	This study
2	${\text{DA}}^{∙-}+O_{2}\overset{k_{2}}{\underset{k_{-2}}{⇄}}\text{DAQ}+O_{2}^{∙-}$	*k*_2 = _2.95 × 10^3^	1
		*k*_−2_ = 1.0 × 10^9^	1
3	${\text{DA}}^{∙-}+{\text{DA}}^{∙-}\overset{k_{3}}{⟶}\text{DA}+\text{DAQ}$	*k*_3_ = 2.35 × 10^8^	2
4	$\text{DAQ}\overset{k_{4}}{⟶}\text{DAL}$	*k*_4_ = 12.7	3
5	$\text{DAL}+\text{DAQ}\overset{k_{5}}{⟶}\text{DA}+\text{DAC}$	*k*_5_ = 5.30 × 10^6^	4
6	$\text{DAL}+O_{2}\overset{k_{6}}{⟶}\text{DAC}+H_{2}O_{2}$	*k*_6_ = 1.62	This study
7	$O_{2}^{∙-}+O_{2}^{∙-}\overset{k_{7}}{⟶}H_{2}O_{2}+O_{2}$	*k*_7_ = 1.90 × 10^5^	5
8	${\text{DA}}^{∙-}+O_{2}^{∙-}\overset{k_{8}}{⟶}\text{DAQ}+H_{2}O_{2}$	*k*_8_ = 8.27 × 10^9^	This study

DA, dopamine; DA^•−^, semiquinone radical; DAQ, dopamine‐*o*‐quinone; $O_{2}^{∙-}$, superoxide; DAC, dopaminochrome; DAL, leukoaminochrome

(1) Pham and Waite (2014); (2) Borovansky *et al*. (2006); (3) Young and Babbitt (1983); (4) Land *et al*. (2003) and (5) Zafiriou (1990).

**Table 2:** Modeled reactions and rate constants for Fe(III)‐catalyzed DA oxidation

No.	Reactions	Rate constants (per Ms or per s)	Reference
9	$\text{Fe(III)}+{\text{Fe(III)}}_{I}\overset{k_{9}}{⟶}\text{AFO}+{\text{nH}}^{+}$	*k*_9_ = 5.0 × 10^6^	6
10	$>{\text{Fe(III)}}_{n}+\text{DA}\overset{k_{10}}{⟶}>{\text{Fe(III)}}_{n-1}+{\text{Fe}}^{\mathrm{III}}\text{DA}$	*k*_10_ = 2.34	This study
11	$>{\text{Fe(III)}}_{n}+\text{DA}\overset{k_{11}}{⟶}>{\text{Fe(III)}}_{n-1}+\text{Fe(II)}+{\text{DA}}^{∙-}$	*k*_11_ = 0.60	This study
12	$\text{Fe(III)}+\text{DA}\overset{k_{12}}{⟶}{\text{Fe}}^{\mathrm{III}}\text{DA}$	*k*_12_ = 2.50 × 10^5^	This study
13	${\text{Fe}}^{\mathrm{III}}\text{DA}+\text{DA}\overset{k_{13}}{⟶}{\text{Fe}}^{\mathrm{III}}{\text{DA}}_{2}$	*k*_13_ = 4.50 × 10^5^	7
14	${\text{Fe}}^{\mathrm{III}}\text{DA}+O_{2}^{∙-}\overset{k_{14}}{⟶}{\text{Fe}}^{\mathrm{II}}\text{DA}+O_{2}$	*k*_14_ = 1.50 × 10^8^	8
15	${\text{Fe}}^{\mathrm{III}}{\text{DA}}_{2}\overset{k_{15}}{⟶}\text{Fe(II)}+\text{DA}+{\text{DA}}^{∙-}$	*k*_15_ = 7.26 × 10^−5^	This study
16	$\text{Fe(III)}+O_{2}^{∙-}\overset{k_{16}}{⟶}\text{Fe(II)}+O_{2}$	*k*_16_ = 1.50 × 10^8^	9
17	$>{\text{Fe(III)}}_{n}+O_{2}^{∙-}\overset{k_{17}}{⟶}>{\text{Fe(III)}}_{n-1}+\text{Fe(II)}+O_{2}$	*k*_17_ = 3.70 × 10^5^	This study
18	$\text{Fe(II)}+{\text{DA}}^{∙-}\overset{k_{18}}{⟶}\text{Fe(III)}+\text{DA}$	*k*_18_ = 1.92 × 10^5^	This study

DA, dopamine; DA^•−^, semiquinone radical; $O_{2}^{∙-}$, superoxide; Fe(II), inorganic ferrous ion; Fe(III), inorganic ferric ion; Fe(III)_I_, total inorganic Fe(III); AFO, amorphous iron oxide.

(6) Pham *et al*. (2006); (7) Blesa and Matijević (1989); (8) Rose and Waite (2003a) and (9) Rush and Bielski (1985).

**Table 3:** Modeled reactions and rate constants for Fe(II)‐catalyzed DA oxidation

No.	Reactions	Rate constants (per Ms or per s)	Reference
19	$\text{Fe(II)}+O_{2}\overset{k_{19}}{⟶}\text{Fe(III)}+O_{2}^{∙-}$	*k*_19_ = 0.77	This study
20	$\text{Fe(II)}+O_{2}^{∙-}\overset{k_{20}}{⟶}\text{Fe(III)}+H_{2}O_{2}$	*k*_20_ = 1 × 10^7^	9	
21	$\text{Fe(II)}+H_{2}O_{2}\overset{k_{21}}{⟶}\text{Fe(III)}{+}^{∙}\text{OH}+{\text{OH}}^{-}$	*k*_21_ = 1.33 × 10^4^	10
22	$\text{Fe}\left(\mathrm{II}\right)+\text{DA}\overset{k_{22}}{⟶}{\text{Fe}}^{\mathrm{II}}\text{DA}$	*k*_22_ = 7.50 × 10^2^	This study
23	${\text{Fe}}^{\mathrm{II}}\text{DA}+O_{2}\overset{k_{23}}{⟶}{\text{Fe}}^{\mathrm{III}}\text{DA}+O_{2}^{∙-}$	*k*_23_ = 1.45 × 10^2^	This study
24	${\text{Fe}}^{\mathrm{II}}\text{DA}+H_{2}O_{2}\overset{k_{24}}{⟶}{\text{Fe}}^{\mathrm{III}}\text{DA}{+}^{∙}\text{OH}+{\text{OH}}^{-}$	*k*_24_ = 1.33 × 10^4^	10
25	${\text{Fe}}^{\mathrm{II}}\text{DA +}\;O_{2}^{∙-}\overset{k_{25}}{⟶}{\text{Fe}}^{\mathrm{III}}\text{DA +}\;H_{2}O_{2}$	*k*_25_ = 1 × 10^7^	8
26	${\text{Fe}}^{\mathrm{II}}\text{DA +}\;{\text{DA}}^{∙-}\overset{k_{26}}{⟶}{\text{Fe}}^{\mathrm{III}}\text{DA + DA}$	*k*_26_ = 1.92 × 10^5^	This study

DA, dopamine; DA^•−^, semiquinone radical; $O_{2}^{∙-}$, superoxide; Fe(II), inorganic ferrous ion; Fe(III), inorganic ferric ion; ^•^OH, hydroxyl radicals.

(10) González‐Davila *et al*. (2005).

## Results

### Autoxidation of DA

**Figure 1:**
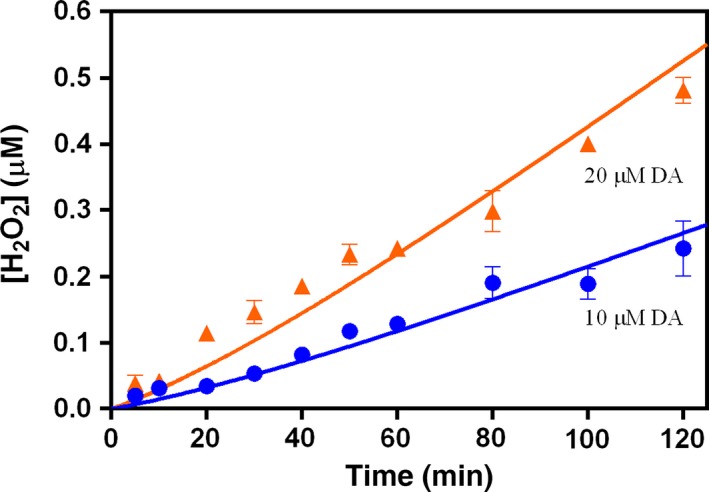
Formation of H_2_O_2_ in the presence of (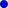) 10 μM and (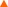) 20 μM dopamine (DA) at pH 7.4 in 0.1 M NaCl. Error bars are standard errors from triplicate measurements and solid lines represent the model fit.

As shown in Fig. 1, the autoxidation of 10 μM and 20 μM DA at pH 7.4 in 0.1 M NaCl generated significant amounts of H_2_O_2_ within 2 h. The generation of H_2_O_2_ is a concentration‐dependent process. At the physiological condition investigated in this study, around 250 nM H_2_O_2_ was generated in the presence of 10 μM DA within 2 h and an almost doubled concentration of H_2_O_2_ (around 500 nM) was generated in the presence of 20 μM DA.

### Interaction of DA with Fe(III)

**Figure 2:**
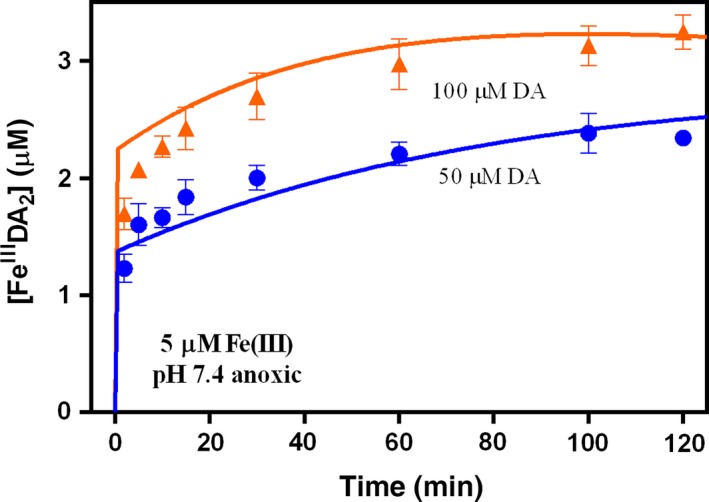
Formation of Fe^III^DA_2_ followed by the addition of 5 μM Fe(III) to deoxygenated solutions containing (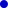) 50 μM and (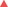) 100 μM dopamine (DA) at pH 7.4 in 0.1 M NaCl. Error bars are standard errors from triplicate measurements and solid lines represent the model fit.

As shown in Fig. 2, in the absence of O_2_, after the addition of Fe(III), very rapid initial formation of Fe^III^DA_2_ followed by a gradual increase in its concentration was observed over the first hour. The concentration of Fe^III^DA_2_ was also found to increase with increase in the DA:Fe(III) concentration ratio. At later times, the increase in Fe^III^DA_2_ concentration was negligible. It was also observed that not all the added Fe(III) was complexed to DA presumably because of the rapid precipitation of Fe(III) at this pH.

**Figure 3:**
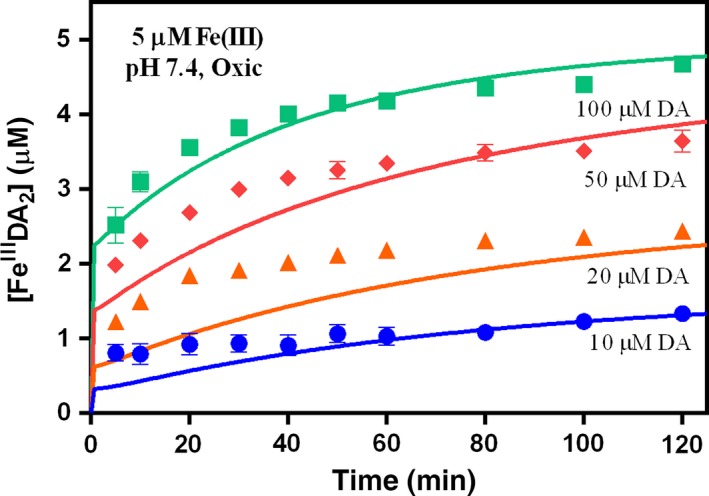
Formation of Fe^III^DA_2_ followed by the addition of 5 μM Fe(III) to oxygen‐saturated solutions containing (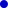) 10 μM, (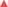) 20 μM, (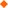) 50 μM and (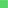) 100 μM dopamine (DA) at pH 7.4 in 0.1 M NaCl. Error bars are standard errors from triplicate measurements and solid lines represent the model fit.

**Figure 4:**
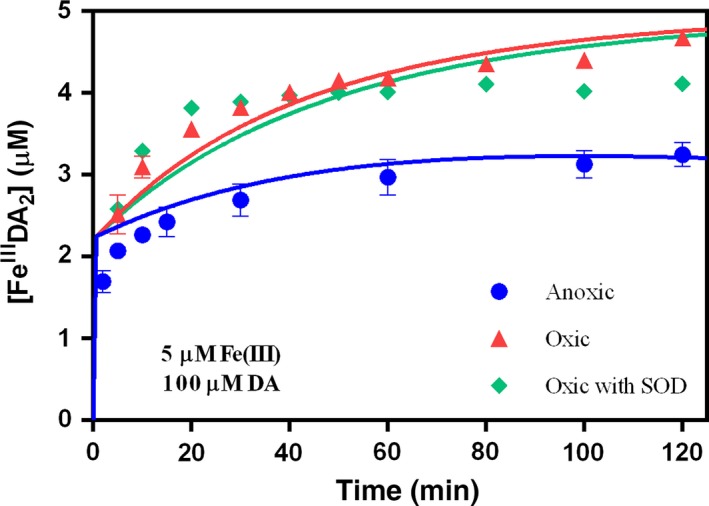
Formation of Fe^III^DA_2_ followed by the addition of 5 μM Fe(III) to solutions containing 100 μM dopamine (DA) at pH 7.4 in 0.1 M NaCl under (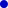) deoxygenated condition, (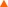) oxygen‐saturated condition and (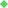) oxygen‐saturated condition with 50 KU/L superoxide dismutase. Error bars are standard errors from triplicate measurements and solid lines represent the model fit.

As shown in Fig. 3, in the presence of O_2_, Fe^III^DA_2_ complex also formed immediately after the addition of Fe(III) into DA containing solutions with a subsequent increase in concentration with increase in DA concentration. However, as shown in Fig. 4, in contrast to the situation in the absence of O_2_, the concentration of Fe^III^DA_2_ complex continued to increase significantly over time, resulting in a substantially higher concentration of Fe^III^DA_2_ complex in the presence of O_2_ at the conclusion of the 2 h study than was the case in the absence of O_2_.

To assess the possible contribution of $O_{2}^{∙-}$to the observed increase in concentration of Fe^III^DA_2_ in the presence of O_2_, 50 KU/L SOD was added to the solution to capture any $O_{2}^{∙-}$produced. As shown in Fig. 4, the addition of SOD only had a minor effect on the concentration of Fe^III^DA_2_.

### Interaction of DA with Fe(II)

**Figure 5:**
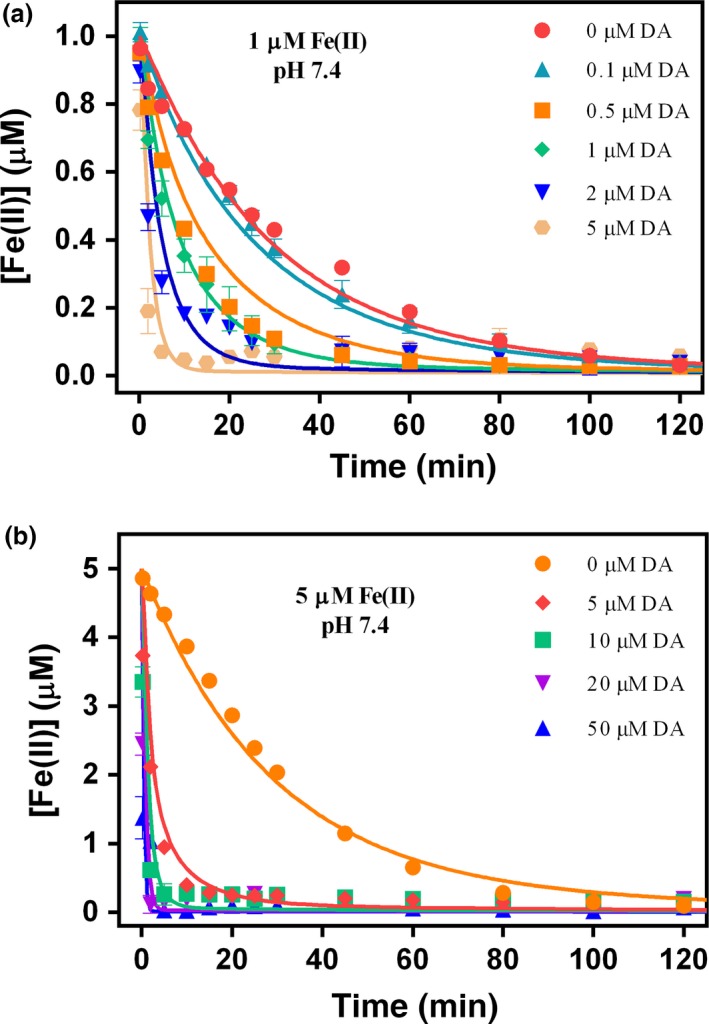
Oxidation of 1 μM (a) and 5 μM (b) Fe(II) in the absence and presence of different concentrations of dopamine (DA) at pH 7.4 in 0.1 M NaCl. Error bars are standard errors from triplicate measurements and solid lines represent the model fit.

As shown in Fig. 5, the rate of oxidation of Fe(II) increased significantly with increase in DA concentration. In the absence of DA, the half‐lives of Fe(II) oxidation were around 25 min at the physiological pH investigated in this study, however, in the presence of DA, the half‐lives can decease to less than 1 min. The ability of DA to induce an increase in the rate of Fe(II) oxidation is consistent with the results of previous catecholamine studies (García *et al*. 2012).

**Figure 6:**
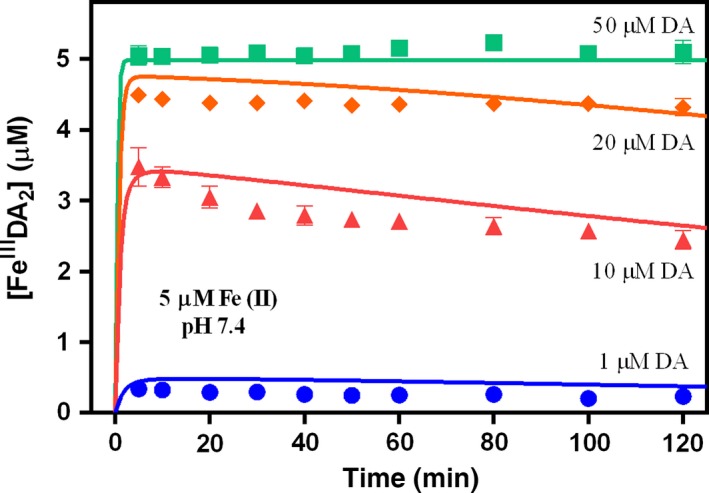
Formation of Fe^III^DA_2_ followed by the addition of 5 μM Fe(II) to oxygen‐saturated solutions containing (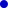) 1 μM, (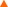) 10 μM, (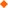) 20 μM and (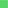) 50 μM DA at pH 7.4 in 0.1 M NaCl. Error bars are standard errors from triplicate measurements.

As shown in Fig. 6, significant amounts of Fe^III^DA_2_ complex were rapidly formed on addition of Fe(II) to solutions containing DA but, unlike the case in which Fe(III) was added to DA‐containing solutions where there was both a rapid initial formation of Fe^III^DA_2_ complex and an ongoing more gradual formation, the concentration of Fe^III^DA_2_ complex gradually decreased following the initial rapid formation on Fe(II) addition. The rapid, initial formation of Fe^III^DA_2_ complex (especially in the presence of 50 μM DA when all the added Fe(II) was converted to Fe^III^DA_2_) confirmed that Fe(II) was first complexed by DA to form Fe^II^DA (reaction 22) with this Fe(II) complex then transforming to the Fe(III) complex through reaction with oxygen (reaction 23). However, the concentration of Fe^III^DA_2_ decreases over time, particularly in the presence of low concentrations of DA (Fig. 6).

**Figure 7:**
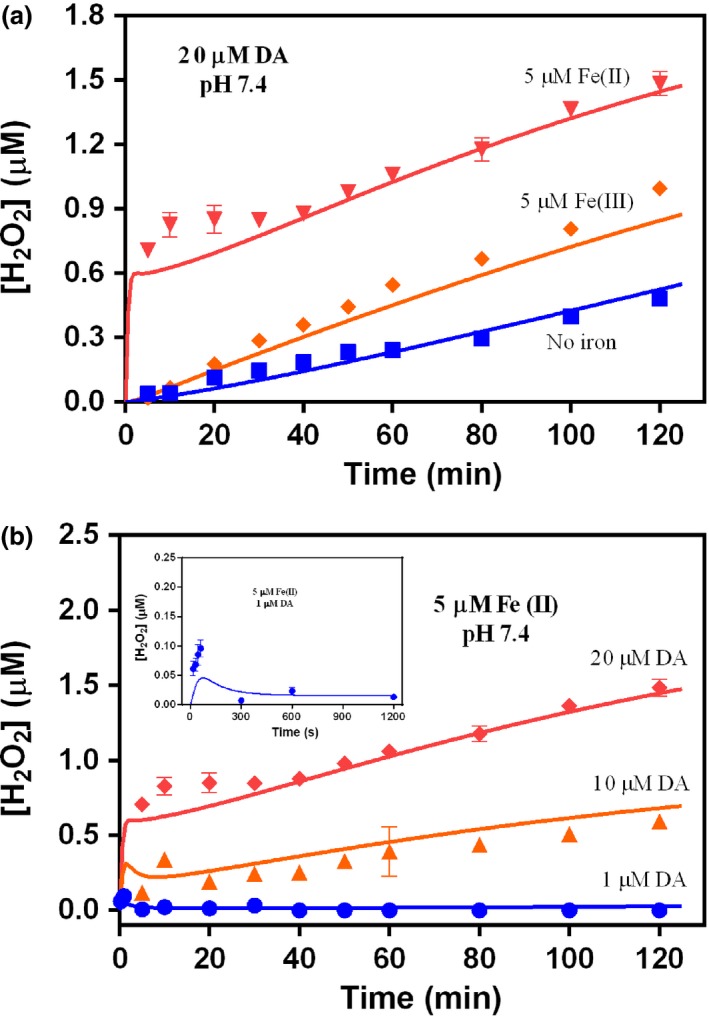
Formation of H_2_O_2_ in oxygen‐saturated solutions at pH 7.4 containing 20 μM dopamine (DA) with (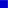) No iron, (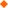) 5 μM Fe(III) only and (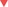) 5 μM Fe(II) only (a) and 5 μM Fe(II) in the presence of (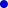) 1 μM, (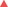) 10 μM and (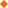) 20 μM DA (b). Error bars are standard errors from triplicate measurements and solid lines represent the kinetic model fit.

The effect of iron‐catalyzed DA oxidation on H_2_O_2_ generation was investigated in view of our interest in the possible formation of ^•^OH via Fenton processes. As shown in Fig. 7(a), the addition of Fe(II) to DA solutions resulted in the generation of significant amounts of H_2_O_2_, especially at the initial stage. H_2_O_2_ generation continued following the initial rapid formation with the concentration of H_2_O_2_ generated by 20 μM DA in the presence of 5 μM Fe(II) more than doubling over the course of the 2 h study. The initial rapid formation of H_2_O_2_ may be attributed to the rapid conversion of Fe(II) to Fe(III) in the presence of high DA concentration as discussed above. However, once Fe(II) was exhausted, the rate of H_2_O_2_ generation after the initial stage (Fig. 7a) was similar to that in the presence of 5 μM Fe(III) and slightly higher than that produced from DA autoxidation alone. The slight increase in the slope of the H_2_O_2_ production in the presence of iron is presumably because of the slow release of Fe(II) from the Fe^III^DA_2_ complex and the subsequent rapid re‐oxidation of Fe(II) to Fe(III) with concomitant reduction in oxygen to superoxide (which, in turn, disproportionates to form H_2_O_2_). Results of DA concentration dependency studies (Fig. 7b) reveal that in the presence of low DA concentration (e.g., 1 μM), almost no H_2_O_2_ can be detected.

## Discussion

### Mechanisms of the interplay between iron and dopamine

As the well‐known neurotransmitter, the oxidation of DA, especially in the presence of iron has been widely studied in the past few decades in view of its potential role in neurotoxicity (Zhang and Dryhurst 1994; Herlinger *et al*. 1995). Even though the pseudo first‐order rate constant of DA autoxidation is small [in the range from 0.03/h to 0.083/h (Herlinger *et al*. 1995; Pham and Waite 2014)] because of the thermodynamic unfavourability (Steenken and Neta 1982; Koppenol *et al*. 2010) and spin restricted nature of this process (Miller *et al*. 1990), there are stoichiometric amounts of H_2_O_2_ generated if oxygen is present. The formation of H_2_O_2_ is generally considered to occur via two one‐electron transfer processes with concomitant consumption of O_2_ and generation of DA^•−^ and DAQ. The generated DAQ generally decays into dopaminochrome (DAC) via the short‐lived cyclization product, leukoaminochrome, which immediately undergoes redox exchange with the remaining DAQ yielding DAC and regenerating DA (Young and Babbitt 1983; Land *et al*. 2003; Borovansky *et al*. 2006). This process, recognized to be pH‐dependent as a result of the need for deprotonation of the amino side chain, is a prerequisite to the subsequent cyclization process (Hawley *et al*. 1967). However, the comproportionation between DA and DAC has been considered unlikely to occur presumably because of the effect of the cyclization of the amino side chain in the DAC (Tse *et al*. 1976; Borovansky *et al*. 2006; Li *et al*. 2007). The proposed reaction scheme for DA autoxidation is presented in Fig. S5. A list of reactions considered likely to be of importance in DA autoxidation together with corresponding rate constants is presented in Table 1.

At the physiological pH investigated in this study, with the co‐existence of iron and DA, the blue‐purple colored Fe^III^DA_2_ complex (shown in Figs S2a, b and c) is the dominant species and is formed through the direct reaction between iron and DA via two sequential steps: an initial rate‐limiting formation of the Fe^III^DA complex (either through the direct reaction between Fe(III) and DA or the oxidation of the Fe^II^DA complex following its formation through the direct reaction between Fe(II) and DA) followed by replacement of one Fe(III)‐coordinated H_2_O by another DA molecule.

In general, there are several processes that may influence the concentration of Fe^III^DA_2_ following its initial formation. Internal electron transfer via the direct reaction between the Fe(III) metal center and the coordinated ligand DA might be expected to be particularly important and will result in loss of Fe^III^DA_2_ with concomitant release of Fe(II) and DA^•−^ (reaction 15) with the rate of decay depending upon the extent of coordination with the mono‐complex being the least stable and the tris‐complex being the most stable (Jameson and Linert 2001). [Note that El‐Avaan *et al*. (1997) reported a rate constant for the internal electron transfer of the mono‐complex Fe^III^DA of 0.23/s while a much smaller constant (of 1.9 × 10^−5^/s) was reported for the monocatecholate complex of Cu(II) (Kamau and Jordan 2002)]. The rate constant for internal electron transfer within the *bis*‐complex Fe^III^DA_2_ (*k*_15_ = 7.26 × 10^−5^/s) reported in this study is undoubtedly in line with this argument. However, if decay via internal electron transfer within the complex is the dominant process controlling the fate of Fe^III^DA_2_, then this should result in a continuous decrease in concentration of Fe^III^DA_2_ following its initial rapid formation both from Fe(III) and Fe(II). This phenomenon, however, is not apparent from the results shown in Figs 2, 3 and 4 which suggests that other sources of Fe^III^DA_2_ are involved including, potentially, the DA‐induced dissolution of any precipitated Fe(III) [initiated by the adsorption of DA to the amorphous ferric oxide (AFO) surface followed by release of Fe^III^DA to solution (reaction 10)] and/or the reductive dissolution of any Fe(III) precipitate [initiated by the adsorption of DA to the AFO surface followed by electron transfer within the Fe(III)‐DA surface complex and subsequent release of Fe(II) (reaction 11)] or even the $O_{2}^{∙-}$ mediated dissolution of AFO. However, as removal of $O_{2}^{∙-}$ by adding SOD did not make much difference in the formation of Fe^III^DA (shown in Fig. 4), these first two pathways may be more important and contribute to the increase in Fe^III^DA_2_ concentration over time.

Model simulations suggest that, under deoxygenated conditions, the reductive dissolution of AFO is not as effective as the ligand (DA)‐induced process for the overall formation of Fe^III^DA_2_ as a result particularly of the apparently rapid back reaction between any released Fe(II) and DA^•−^ (reaction 18). The re‐oxidation of the released Fe(II) by DA^•−^ exerts significant influence on the formation of Fe^III^DA_2_, especially at the later stage of the reaction. In contrast to the deoxygenated condition, in the presence of O_2_, both the rapid oxidation of Fe(II) at pH 7.4 (reaction 19), which directly contributes to the generation of Fe(III) (and thus of Fe^III^DA_2_), and the oxidation of DA^•−^ by O_2_ drive the reductive dissolution reaction to the right, making the reductive dissolution of AFO be the main factor contributing to the increase in concentration of Fe^III^DA_2_.

As shown in Figs 2, 3 and 6, no matter in the presence of Fe(III) or Fe(II), the concentration of Fe^III^DA_2_ complex increased significantly with increasing DA concentration. The increase in concentration of the Fe^III^DA_2_ complex with increasing DA concentration in the absence and presence of O_2_ is reasonable since a higher concentration of DA would be more effective in preventing Fe(III) precipitation and accelerating Fe(II) oxidation. At high DA concentration (e.g., 50 μM), presumably, any Fe(III) existing in the solution or released from the complex (as a result of dissociation of the complex) or any Fe(II) existing in the solution or released from the complex (as a result of internal electron transfer) would be rapidly re‐bound (in the case of Fe(II), following reoxidation) by DA. In comparison, at low DA concentrations, Fe(III) hydrolysis may compete with complexation resulting in the formation of AFO rather than Fe^III^DA_2_. Similarly, the oxidation of any existing or released Fe(II) may result in formation of AFO rather than Fe^III^DA_2_ if there is insufficient DA to compete with the hydrolysis reaction. The gradual decrease in the concentration of Fe^III^DA_2_ observed at 20, 10, and 1 μM DA (Fig. 6) can most likely be accounted for by the slow replacement of Fe^III^DA_2_ by AFO as a result of the inability of complexation to outcompete hydrolysis at these lower DA concentrations.

In the presence of DA, the oxidation of Fe(II) becomes more thermodynamically favorable as the formation of the colorless mono‐complex with Fe(II) (Powell and Taylor 1982; Perron and Brumaghim 2009) facilitates the electron transfer between Fe(II) and O_2_ by lowering the reduction potential of the Fe(III)/Fe(II) half‐reaction. Details of the expected change in the standard redox potential of the Fe(III)/Fe(II) half‐reaction are shown in the supplementary information (Text S1).

As a transition metal with several spin states, iron can catalyze the oxidation of DA and accelerate the generation of H_2_O_2_ by ligating its *d* orbitals to both O_2_ and DA, thereby effectively serving as a bridge between O_2_ and DA with resultant elimination of the spin restricted nature of the interaction between these two molecules. However, the co‐existence of excess Fe(II) with H_2_O_2_ generated from both DA autoxidation and iron‐catalyzed oxidation of DA may result in the generation of ^•^OH through ‘Fenton chemistry’. As shown in Figs 5 and 7, considering the relatively slow oxidation kinetics of Fe(II) in the presence of low DA concentrations (e.g., 1 μM), any H_2_O_2_ that was produced in the process may react quickly with the remaining Fe(II) via the Fenton reaction to generate Fe(III) and the powerful oxidizing species ^•^OH. However, in the presence of high DA concentrations, as shown in Figs 5 and 6, almost all Fe(II) was converted into Fe^II^DA and then Fe^III^DA_2_ immediately upon addition. Thus, even though significant concentrations of H_2_O_2_ were generated in the presence of Fe(II), the Fenton reaction is unlikely to be important under these conditions as the majority of Fe(II) was locked in the Fe^III^DA_2_ complex before it had a chance to react with the large amount of H_2_O_2_ generated during this process. This is also in agreement with the proposed protective role of phenol‐type compounds in view of their ability to bind Fe(II) with resultant prevention of the reaction of Fe(II) with H_2_O_2_, ^•^OH production and subsequent cell and DNA damage (Perron *et al*. 2008, 2010; Perron and Brumaghim 2009). A dual role of L‐DOPA in which it may act as a pro‐oxidant at low concentrations and an anti‐oxidant at high concentrations has also been proposed (Spencer *et al*. 1996). However, it is likely that, in the long term, accumulation of high concentrations of intracellular DA may also be deleterious as the pro‐oxidant role of DA is H_2_O_2_ concentration‐dependent and, once the accumulation of H_2_O_2_ is high enough, peroxide‐mediated oxidation of Fe(II) (with associated ^•^OH production) would almost certainly outcompete the oxygen‐mediated oxidation of Fe(II) (a pathway which does not result in production of ^•^OH).

As the presence of excess neural labile iron is detrimental and may cause biological damage as a result of the generation of ROS, a chelation strategy may help to reduce the oxidative damage associated with regional iron deposition. Indeed, a variety of chelators have been investigated for the rectification of disorders related to iron‐overload over the past few decades (Gassen and Youdim 1997; Hider *et al*. 2011; Mounsey and Teismann 2012). For example, treatment of patients in clinical trials with chelators such as deferiprone and clioquinol has been found to be effective in delaying the progression of both PD (Devos *et al*. 2014) and Alzheimer's disease (Kaur *et al*. 2003). Considering the medically meaningful concentrations of both dopamine and iron used in this study and the generally good fit of the model to the obtained data, it would seem reasonable to suggest that the model developed in this study could be of assistance in predicting the appropriate dosages of chelators that should be used in future clinical trials.

### Modeling the kinetics of DA oxidation in the absence and presence of iron

Results of model fitting using the reaction scheme presented in Tables 1, 2, 3 are shown for (i) generation of H_2_O_2_ arising from DA oxidation both in the absence (Fig. 1) and presence of Fe(II) and Fe(III) (Fig. 7); (ii) formation of Fe^III^DA_2_ following the addition of Fe(III) (Figs 2 and 3) and Fe(II) (Fig. 6) to solutions containing varying concentrations of DA in the absence and presence of O_2_ and (iii) the effect of DA on Fe(II) oxidation (Fig. 5). Rate constants for the various reactions that have been used in the model fitting are summarized in Tables 1, 2, 3. Discussion of factors underpinning the selection of each rate constant is provided below as are results of sensitivity analyses used to determine the importance of the various proposed reactions.

From a thermodynamic perspective, the oxidation of DA by both $O_{2}^{∙-}$ and H_2_O_2_ is energetically unfavorable because *E* ^0^(DA^•−^/DA) = 1.353 V > *E* ^0^($O_{2}^{∙-}$/H_2_O_2_) = 1.05 V and *E* ^0^(H_2_O_2_/^•^OH) = 0.39 V (Koppenol *et al*. 2010; Pham and Waite 2014). In addition, sensitivity analysis of the rate constant of the reaction between DA and $O_{2}^{∙-}$ (Fig. S4a) indicated that the oxidation of DA by $O_{2}^{∙-}$ was only important when the rate constant of this reaction is over 1 × 10^3^/M/s. Therefore, the oxidation of DA by both $O_{2}^{∙-}$ and H_2_O_2_ were not considered in the reaction scheme. In contrast, the oxidation of DA^•−^ by $O_{2}^{∙-}$ is much more energetically favourable because *E* ^0^($O_{2}^{∙-}$/H_2_O_2_) = 1.05 V > *E* ^0^(DAQ/DA^•−^) = 0.15 V (Pham and Waite 2014) and exerted significant influence on the formation of *bis*‐Fe^III^DA_2_ complex in the presence of O_2_ at the later stage of the reaction. The rate constant used in this study for the redox exchange between leukoaminochrome and DAQ (*k*_5_ = 5.30 × 10^6^/M/s) was adopted from a previous dopaquinone study in view of the similarity in structure between dopamine and dopaquinone (Land *et al*. 2003). As shown in Fig. S4b, only an upper limit of 1.0 × 10^7^/M/s for this rate constant is deduced from sensitivity analysis. Furthermore, addition of a reaction depicting the formation of melanin from DAC resulted in no change in the relative residual *r* when the rate constant for this reaction was varied from 10^−3^ to 10^9^/M/s (Fig. S4b). This result suggests that the formation of melanin from DAC was not important in the early stage of the DA oxidation and thus was not included in the proposed model.

In the presence of DA, as shown in Fig. S4c, both the ligand‐induced dissolution of AFO as a result of DA adsorption onto the solid surface (reaction 10) and the DA‐induced reductive dissolution of AFO as a result of internal electron transfer (reaction 11) were important processes as the relative residual *r* was quite sensitive to change in the magnitude of each rate constant. On the other hand, only an upper value of 7.26 × 10^−5^/s (Fig. S4d) was deduced for internal electron transfer between Fe(III) and the coordinated DA within the *bis*‐Fe^III^DA_2_ complex (reaction 15).

To have a better understanding of the formation rate constant of the rate‐limiting Fe^III^DA complex from the direct reaction between Fe(III) and DA (reaction 12), the initial concentrations of the precipitate (amorphous iron oxide, AFO) and of Fe^III^DA_2_ thus were used to estimate crudely the formation rate constant of the Fe^III^DA complex by using the published Fe(III) precipitation rate constant (5.0 × 10^6^/M/s) (Pham *et al*. 2006) and the assumption that only precipitation (reaction 9) and complexation (reaction 12) of Fe(III) control the concentration of Fe(III) initially. Thus,

(1) $$\frac{d\left[\text{AFO}\right]}{dt}=k_{9}\left[\text{Fe}\left(\text{III}\right)\right]\left[\text{Fe}{\left(\text{III}\right)}_{I}\right]$$

(2) $$\frac{d\left[{\text{Fe}}^{\mathrm{III}}\text{DA}\right]}{dt}=k_{12}\left[\text{Fe}\left(\text{III}\right)\right]\left[\text{DA}\right]$$

where Fe(III)_I_ is the total inorganic Fe(III) (including both dissolved and precipitated ferric species).

Given that [DA] is in considerable excess of total ferric ion concentration ([Fe(III)]_T_), i.e. [DA] ≈ [DA]_T_, substituting [Fe(III)_I_] = [Fe(III)]_T_ − [Fe^III^DA] and [Fe(III)] = [Fe(III)]_T_ − [Fe^III^DA] − [AFO], the rate laws may be written as:

(3) $$\begin{array}{cc} \frac{d\left[\text{AFO}\right]}{dt} & =k_{9}\left({\left[\text{Fe}\left(\text{III}\right)\right]}_{T}-\left[{\text{Fe}}^{\mathrm{III}}\text{DA}\right]-\left[\text{AFO}\right]\right)\\ & \;\left({\left[\text{Fe}\left(\text{III}\right)\right]}_{T}-\left[{\text{Fe}}^{\mathrm{III}}\text{DA}\right]\right) \end{array}$$

(4) $$\frac{d\left[{\text{Fe}}^{\mathrm{III}}\text{DA}\right]}{dt}=k_{12}^{\prime }\left({\left[\text{Fe}\left(\text{III}\right)\right]}_{T}-\left[{\text{Fe}}^{\mathrm{III}}\text{DA}\right]-\left[\text{AFO}\right]\right)$$

where $k_{12}^{\prime }=k_{12}{\left[\text{DA}\right]}_{T}$. Solution for this linear OED is of the form (Pham *et al*. 2006)

(5) $$\frac{{\left[\text{Fe}\left(\text{III}\right)\right]}_{T}}{{\left[{\text{Fe}}^{\mathrm{III}}\text{DA}\right]}_{\mathrm{eq}}}=\frac{k_{9}}{k_{12}}\times \frac{2{\left[\text{Fe}\left(\text{III}\right)\right]}_{T}-{\left[{\text{Fe}}^{\mathrm{III}}\text{DA}\right]}_{\mathrm{eq}}}{2{\left[\text{DA}\right]}_{T}}+1$$

where [Fe^III^DA]_eq_ is the concentration of [Fe^III^DA] at the initial pseudo‐equilibrium. Fe^III^DA, which instantly converted to Fe^III^DA_2_, can be spectrophotometrically measured at 570 nm.

At 50 μM DA and 5 μM Fe(III), substituting [Fe(III)]_T_ = 5 μM, [DA]_T_ = 50 μM, *k*_9_ = 5.0 × 10^6^/M/s and [Fe^III^DA]_eq_ ≈ [Fe^III^DA_2_] = 1.23 μM to equation 5 gives *k*_12_ = 1.43 × 10^5^/M/s. Similarly, at 100 μM DA and 5 μM Fe(III), substituting [Fe(III)]_T_ = 5 μM, [DA]_T_ = 100 μM, *k*_9_ = 5.0 × 10^6^/M/s and [Fe^III^DA]_eq_ ≈ [Fe^III^DA_2_] = 1.70 μM into equation 5 gives *k*_12_ = 1.07 × 10^5^/M/s. These approximate values were used as a constraint for the model fitting of *k*_12_.

Based on the analysis above and sensitivity analysis (Fig. S4e), a best‐fit rate constant of 2.50 × 10^5^/M/s was deduced for formation of the mono‐complex Fe^III^DA (reaction 12). This value is consistent with the formation rate constants proposed for the complexation of a range of natural organic ligands with Fe(III) (Rose and Waite 2003b). The rate constant for the formation of the *bis*‐complex Fe^III^DA_2_ from Fe^III^DA and DA (reaction 13) is assumed to be similar to the rate constant for water‐loss from Fe(OH)(H_2_O)_5_^2+^ of 4.50 × 10^5^/M/s (Blesa and Matijević 1989) in view of the fact that the replacement of a coordinated H_2_O by another DA molecule is generally faster than the formation of the mono‐complex (Ludwig *et al*. 1995). The results of sensitivity analyses shown in Fig. S4e however indicate that this assigned value should be treated as a lower limit since variation of *k*_13_ above this value did not result in any significant variation in the relative residual *r*. As shown in Fig. S4f, the best‐fit rate constant for the reaction between $O_{2}^{∙-}$ and AFO of 3.70 × 10^5^/M/s (reaction 17) is almost three orders of magnitude less than that with dissolved inorganic Fe(III) (of 1.50 × 10^8^/M/s). Given that the reactivity of $O_{2}^{∙-}$ with AFO is strongly dependent upon the age and structure of AFO (Fujii *et al*. 2008), the significant difference between these two rate constants is not unexpected. Introducing a reaction between AFO and DA^•−^ (to form Fe(II) and DAQ) with a rate constant varying from 10^−2^ to 10^6^/M/s (Fig. S4f) did not vary the relative residual *r* with this result suggesting that this reaction was unimportant and, as such, was not included in the model.

As a result of both a lower charge and a larger radius, Fe(II) is expected to form complexes with DA more slowly than with Fe(III) (Uchimiya and Stone 2006) and this prediction is in accord with results of this study. The rate constant for formation of a complex between Fe(II) and DA deduced in this study of 7.50 × 10^2^/M/s (reaction 22 with sensitivity analysis given in Fig. S4g) is consistent with values proposed for Fe(II) complexation with a range of naturally occurring organic compounds (Rose and Waite 2003b). The dissociation rate constants of the mono‐complexes Fe^II^DA and Fe^III^DA calculated from their corresponding equilibrium constants (Table S1) and formation rate constants (Tables 2 and 3) are ~ 10^−6^ and ~ 10^−16^/s, respectively, which are too small to be significant. Thus, the dissociation reactions of these complexes were not considered in this study.

It is clear from the results obtained that oxidative intermediates DA^•−^, $O_{2}^{∙-}$ and H_2_O_2_ play an important role in the transformation of both inorganically and organically complexed Fe(II) and Fe(III). However, because of the considerable insensitivity of the relative residuals to variation in the rate constants of the reactions between organically complexed Fe(II) with DA^•−^, $O_{2}^{∙-}$ and H_2_O_2_ (Fig. S4e, g and h) and the reduction in organically complexed Fe(III) with $O_{2}^{∙-}$ (Fig. S4h), these rate constants were assumed to be of similar order of magnitude to that for reaction of these species with inorganic iron. In contrast to the reactions with $O_{2}^{∙-}$ as shown in Fig. S4h, reduction in both inorganically and organically complexed Fe(III) with DA^•−^ is not important given the insensitivity of the relative residuals to variation in the rate constants (from 1 to 10^9^/M/s) of these reactions. Therefore, the reduction in inorganically and organically complexed Fe(III) by DA^•−^ was not considered in the proposed reaction scheme.

## Conclusions and implications

The results of this study show that, in the presence of O_2_, a considerable amount of H_2_O_2_ can be generated during the autoxidation of DA with this process significantly accelerated in the presence of both Fe(III) and Fe(II). In the presence of Fe(III), rapid formation of the *bis*‐complex Fe^III^DA_2_ at physiological pH followed by slow internal electron transfer within the complex results in production of Fe(II) and DA^•−^ which, in the presence of oxygen, are oxidized with concomitant formation of superoxide. The superoxide so formed then either disproportionates or is further reduced, resulting in production of H_2_O_2_ in addition to that produced by DA alone. Observation of the initial increase in concentration of Fe^III^DA_2_ in the absence and presence of oxygen suggested that mobilization of iron from amorphous ferric oxide by DA through both ligand‐induced dissolution and reductive processes was operating at the physiological pH used in the studies described here. As DA is more effective in inducing AFO dissolution in the presence of oxygen, we conclude that the reductive mechanism of DA‐mediated AFO dissolution predominates. Observation that addition of SOD exerted a minor influence on the formation of Fe^III^DA_2_ suggested that $O_{2}^{∙-}$ ‐induced Fe(III) dissolution from AFO may not be as important as previously thought.

In the presence of Fe(II), instantaneous formation of the mono‐complex Fe^II^DA followed by reaction with O_2_ resulted in conversion of almost all Fe(II) present in this complex into the more stable Fe^III^DA_2_ complex at high DA concentrations. While this direct reaction between Fe(II) and DA resulted in rapid generation of a substantial amount of H_2_O_2_, the deleterious effect of reaction of this H_2_O_2_ with Fe(II) (the ‘Fenton reaction’) was avoided as the majority of the Fe(II) present was rapidly converted to Fe^III^DA_2_ via an oxygenation pathway. In comparison, at low DA concentrations, a slow oxygenation rate of Fe(II) coupled with a rapid depletion of generated H_2_O_2_ in the presence of excess iron suggested that Fe(II) might react with H_2_O_2_ to form strongly oxidizing ^•^OH. Even though the oxidation of Fe(II) by O_2_ may still be important, the presence of additional H_2_O_2_ as a result of the presence of DA provides a pathway for the formation of strongly oxidizing ^•^OH.

A relatively simple kinetic model has been developed and found to satisfactorily describe the overall kinetics of generation of H_2_O_2_ through the oxidation of DA in the absence and presence of Fe(II) and Fe(III), the formation of Fe^III^DA_2_ complex in the presence of Fe(III) and Fe(II) in both oxygenated and deoxygenated environments and the oxidation of Fe(II) in the presence of DA over a range of DA/iron ratios at physiological pH. While there are many additional factors that would need to be considered in extending this model to description of *in vivo* intracellular processes, this model should assist in better understanding the anti‐oxidant and pro‐oxidant properties of intracellular DA, especially with regard to its role in the transformation of intracellular iron. In addition, the mechanistically based kinetic model developed here should aid in predicting the appropriate dosages of iron chelators that could be used in clinical trials aimed at examining the efficacy of chelation therapy to treatment of neurodegenerative disorders such as Parkinson's disease.

## Supporting information


**Table S1.** Stability constants for Fe(II) and Fe(III) speciations at 25°C and *I *= 0.
**Figure S1.** Measured absorbance of H_2_O_2_ at 551 nm with baseline corrected at 690 nm in 0.1 M NaCl at pH 7.4 in the absence of Fe(III) and DA (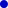) and in the presence of 5 μM Fe(III) (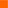) and 20 μM DA (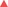). Error bars are standard errors from triplicate measurements.
**Figure S2.** Speciation of Fe(III) (a, b and c) and Fe(II) (d, e and f) over the pH range 4.0–8.5 in solutions containing varying concentrations of DA with [Fe(II)]_0_ = 5 μM and [Fe(III)]_0_ = 5 μM. LogC denotes the log concentration of individual ferrous and ferric species.
**Figure S3.** Measured absorbance of Fe^III^DA_2_ complexes (a) and calibration curve for qualification of Fe^III^DA_2_ complexes measured at 570 nm with baseline corrected at 850 nm (b) in 0.1 M NaCl at pH 7.4 in the presence of 400 μM DA under deoxygenated condition.
**Figure S4.** Sensitivity analysis for different fitting reaction rate constants (Tables 1, 2, 3, main text) and some other unimportant reactions.
**Figure S5.** Oxidation pathway of dopamine in the absence of added metals.
**Text S1.** Effect of DA on the standard redox potential of the Fe(III)/Fe(II) half‐reaction.
