# Supplementary material for: Elucidation of the interplay between Fe(II), Fe(III), and dopamine with relevance to iron solubilization and reactive oxygen species generation by catecholamines
Source: J Neurochem. 2016 May 12;137(6):955–68. doi: 10.1111/jnc.13615 (PMC6191651; doi:10.1111/jnc.13615)
Supplement: Supplementary file 1 — Table S1. Stability constants for Fe(II) and Fe(III) speciations at 25°C and I = 0. Figure S1. Measured absorbance of H2O2 at 551 nm with baseline corrected at 690 nm in 0.1 M NaCl at pH 7.4 in the absence of Fe(III) and DA () and in the presence of 5 μM Fe(III) () and 20 μM DA (). Error bars are standard errors from triplicate measurements. Figure S2. Speciation of Fe(III) (a, b and c) and Fe(II) (d, e and f) over the pH range 4.0–8.5 in solutions containing varying concentrations of DA with [Fe(II)]0 = 5 μM and [Fe(III)]0 = 5 μM. LogC denotes the log concentration of individual ferrous and ferric species. Figure S3. Measured absorbance of FeIIIDA2 complexes (a) and calibration curve for qualification of FeIIIDA2 complexes measured at 570 nm with baseline corrected at 850 nm (b) in 0.1 M NaCl at pH 7.4 in the presence of 400 μM DA under deoxygenated condition. Figure S4. Sensitivity analysis for different fitting reaction rate constants (Tables 1, 2, 3, main text) and some other unimportant reactions. Figure S5. Oxidation pathway of dopamine in the absence of added metals. Text S1. Effect of DA on the standard redox potential of the Fe(III)/Fe(II) half‐reaction. [file JNC-137-955-s001.pdf]

Supporting Information for the Manuscript Entitled

Elucidation of the Interplay between Fe(II), Fe(III) and Dopamine with Relevance to Iron Solubilization and ROS Generation by Catecholamines

Yingying Sun, A. Ninh Pham and T. David Waite*

School of Civil and Environmental Engineering, The University of New South Wales, Sydney, NSW 2052, Australia

Running title: Iron, dopamine and ROS

Re-submitted

Journal of Neurochemistry

February, 2016

* Corresponding author. Professor T. David Waite, Tel.: +61 2 9385 5059, Email: d.waite@unsw.edu.au

Table S1. Stability constants for Fe(II) and Fe(III) speciations at 25 ℃ and I = 0.

No.	species	Log*K*	Reference
Fe(II) species			
1	$Fe^{2+} + H_2O \rightleftharpoons FeOH^{+} + H^{+}$	-9.51	1
2	$Fe^{2+} + 2H_2O \rightleftharpoons Fe(OH)_2^0 + 2H^{+}$	-20.6	1
3	$Fe^{2+} + CO_3^{2-} \rightleftharpoons FeCO_3^0$	5.69	2
4	$Fe^{2+} + H^{+} + CO_3^{2-} \rightleftharpoons FeHCO_3^{+}$	11.8	3
5	$Fe^{2+} + 2CO_3^{2-} \rightleftharpoons Fe(CO_3)_2^{2-}$	7.45	2
6	$Fe^{2+} + CO_3^{2-} + H_2O \rightleftharpoons Fe(OH)CO_3^{-} + H^{+}$	-4.03	2
7	$Fe^{2+} + Cl^{-} \rightleftharpoons FeCl^{+}$	0.3	2
8	$Fe^{2+} + SO_4^{2-} \rightleftharpoons FeSO_4^0$	2.42	2
9	$Fe^{2+} + DA^{2-} \rightleftharpoons FeDA^0$	9.12	4
10	$Fe^{2+} + 2DA^{2-} \rightleftharpoons FeDA_2^{2-}$	14.56	4
Fe(III) species			
11	$Fe^{3+} + H_2O \rightleftharpoons Fe(OH)^{2+} + H^{+}$	-2.13	5
12	$Fe^{3+} + 2H_2O \rightleftharpoons Fe(OH)_2^{+} + 2H^{+}$	-6.13	5
13	$Fe^{3+} + 3H_2O \rightleftharpoons Fe(OH)_3^0 + 3H^{+}$	-14.3	5
14	$Fe^{3+} + 4H_2O \rightleftharpoons Fe(OH)_4^{-} + 4H^{+}$	-22.2	5
15	$Fe^{3+} + Cl^{-} \rightleftharpoons FeCl^{2+}$	1.28	5
16	$Fe^{3+} + 2Cl^{-} \rightleftharpoons FeCl_2^{+}$	1.16	5
17	$Fe^{3+} + SO_4^{2-} \rightleftharpoons Fe(SO_4)^{+}$	4.27	5

18	$Fe^{3+} + 2SO_4^{2-} \rightleftharpoons Fe(SO_4)_2^-$	6.11	5
19	$Fe^{3+} + 2CO_3^{2-} \rightleftharpoons Fe(CO_3)_2^-$	19.6	5
20	$Fe^{3+} + DA^{2-} \rightleftharpoons FeDA^+$	21.42	6
21	$Fe^{3+} + 2DA^{2-} \rightleftharpoons FeDA_2^-$	36.46	6
22	$Fe^{3+} + 3DA^{2-} \rightleftharpoons FeDA_3^{3-}$	45.08	6
Aqueous species			
23	$H^+ + OH^- \rightleftharpoons H_2O$	14	7
24	$H^+ + CO_3^{2-} \rightleftharpoons HCO_3^-$	10.3	7
25	$2H^+ + CO_3^{2-} \rightleftharpoons H_2CO_3^*$	16.7	7
26	$NH_3 + H^+ \rightleftharpoons NH_4^+$	9.24	8
27	$H^+ + SO_4^{2-} \rightleftharpoons HSO_4^-$	1.99	8
28	$Na^+ + CO_3^{2-} \rightleftharpoons NaCO_3^-$	1.27	2
29	$Na^+ + H^+ + CO_3^{2-} \rightleftharpoons NaHCO_3^0$	10.1	2
30	$Na^+ + SO_4^{2-} \rightleftharpoons NaSO_4^-$	1.06	2
31	$NH_4^+ + SO_4^{2-} \rightleftharpoons NH_4SO_4^-$	1.03	9
32	$H_2DA \rightleftharpoons HDA^- + H^+$	-10.58	10
33	$HDA^- \rightleftharpoons DA^{2-} + H^+$	-12.07	10

(1) Morel and Hering (1993); (2) King (1998); (3) Millero and Hawke (1992); (4) Smith and Martell (1989); (5) Pham *et al.* (2006); (6) Avdeef *et al.* (1978); (7) Millero et al. (1995); (8) Yuan *et al.* (2012); (9) Schecher and McAvoy (1992) and (10) Pham and Waite (2014)

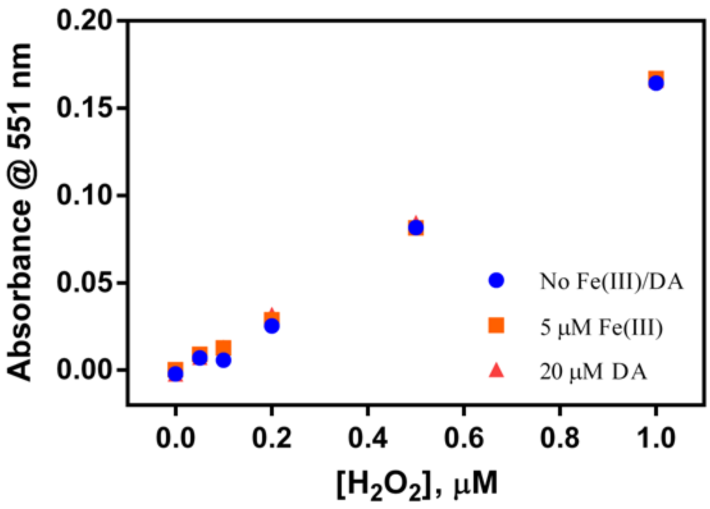

Figure S1. Measured absorbance of H_2O_2 at 551 nm with baseline corrected at 690 nm in 0.1 M NaCl at pH 7.4 in the absence of Fe(III) and DA (●) and in the presence of 5 µM Fe(III) (■) and 20 µM DA (▲). Error bars are standard errors from triplicate measurements.

Figure S1 indicated that the presence of moderate concentrations of Fe(III) (5 µM) and DA (20 µM) has negligible effect on the measurement of H_2O_2 in the presence of 1 mM DTPA, 60 µM DPD and 500 U/L HRP .

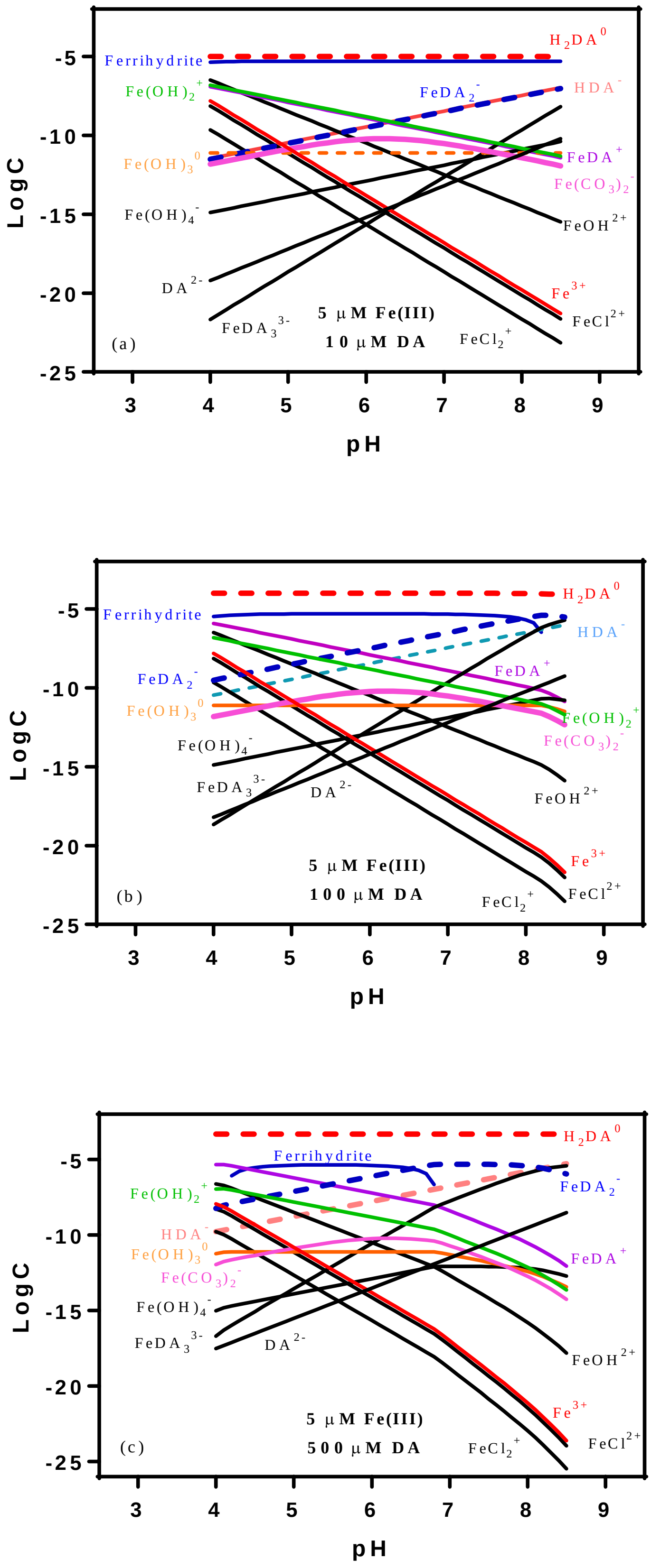

LogC
pH
Ferrihydrite
H_2DA^0
HDA^-
$FeDA_2^-$
$Fe(OH)_2^+$
$Fe(OH)_3^0$
$Fe(OH)_4^-$
$FeDA^+$
$Fe(CO_3)_2^-$
$FeOH^{2+}$
DA^{2-}
$FeDA_3^{3-}$
Fe^{3+}
$FeCl^{2+}$
$FeCl_2^+$
(a)
5 μM Fe(III)
10 μM DA
(b)
5 μM Fe(III)
100 μM DA
(c)
5 μM Fe(III)
500 μM DA

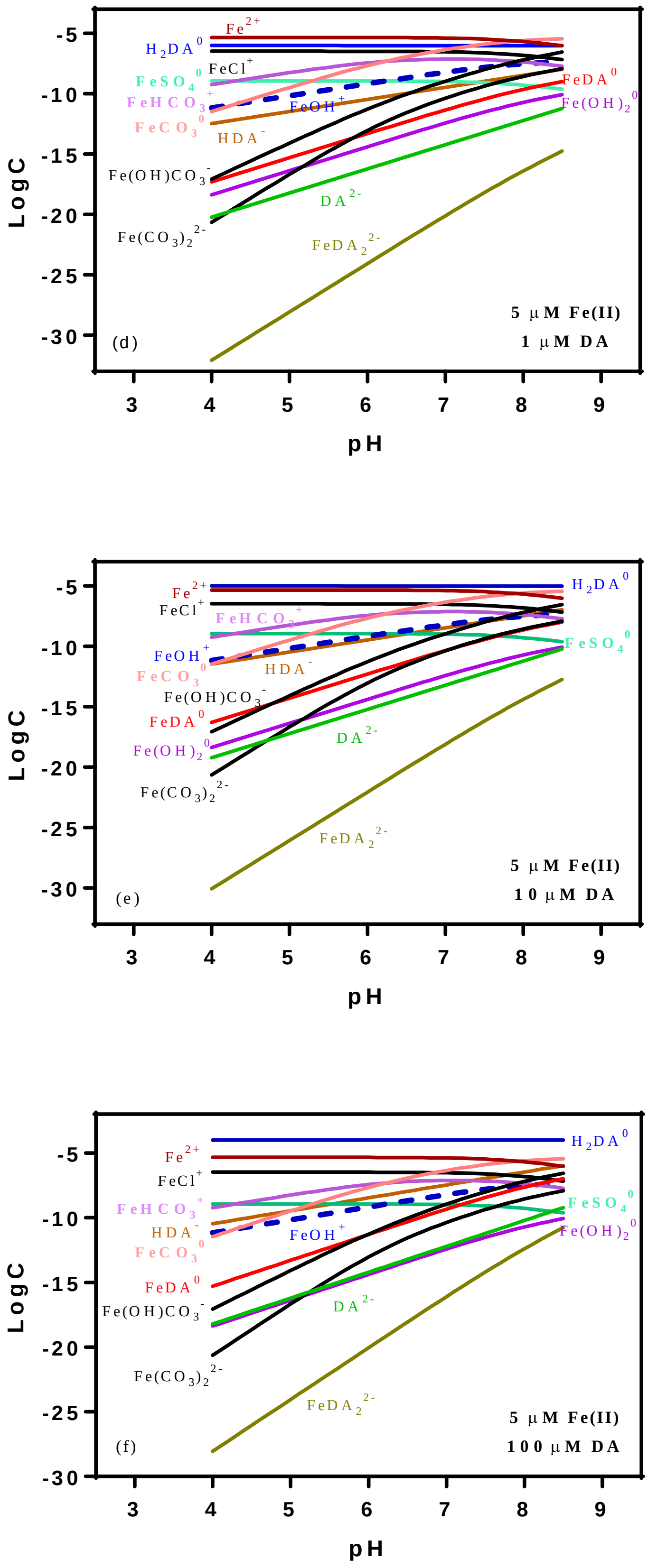
Fe2+
H2DA0
FeCl+
FeSO40
FeHCO3+
FeCO30
FeOH+
HDA-
FeDA0
Fe(OH)20
Fe(OH)CO3-
DA2-
Fe(CO3)22-
FeDA22-
5 μM Fe(II)
1 μM DA
(d)
5 μM Fe(II)
10 μM DA
(e)
5 μM Fe(II)
100 μM DA
(f)
LogC
pH
-5
-10
-15
-20
-25
-30
3
4
5
6
7
8
9

Figure S2. Speciation of Fe(III) (a, b and c) and Fe(II) (d, e and f) over the pH range 4.0 - 8.5 in solutions containing varying concentrations of DA with $[Fe(II)]_0$ = 5 μM and $[Fe(III)]_0$ = 5 μM. LogC denotes the log concentration of individual ferrous and ferric species.

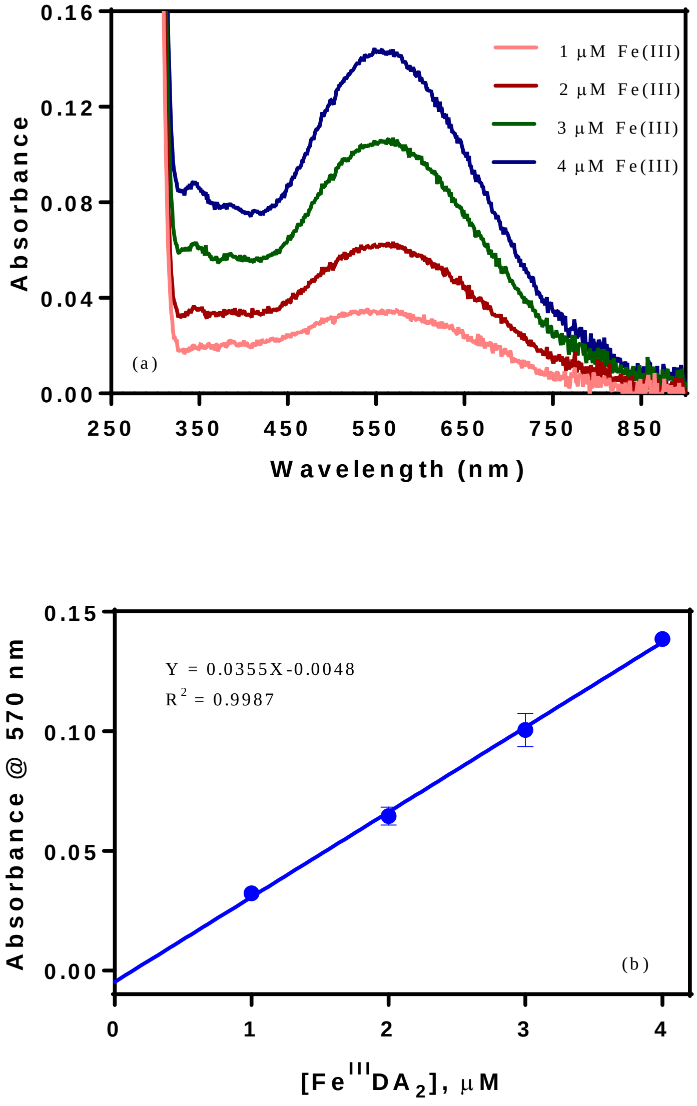

Figure S3. Measured absorbance of $Fe^{III}DA_2$ complexes (panel a) and calibration curve for qualification of $Fe^{III}DA_2$ complexes measured at 570 nm with baseline corrected at 850 nm (panel b) in 0.1 M NaCl at pH 7.4 in the presence of 400 μM DA under deoxygenated condition.

Figure S3 indicated that in the presence of moderate concentrations of DA, $Fe^{III}DA_2$ was the dominant complexes and a clear peak can be observed at 570 nm. 400 μM DA was used to ensure that precipitation of Fe(III) is negligible.

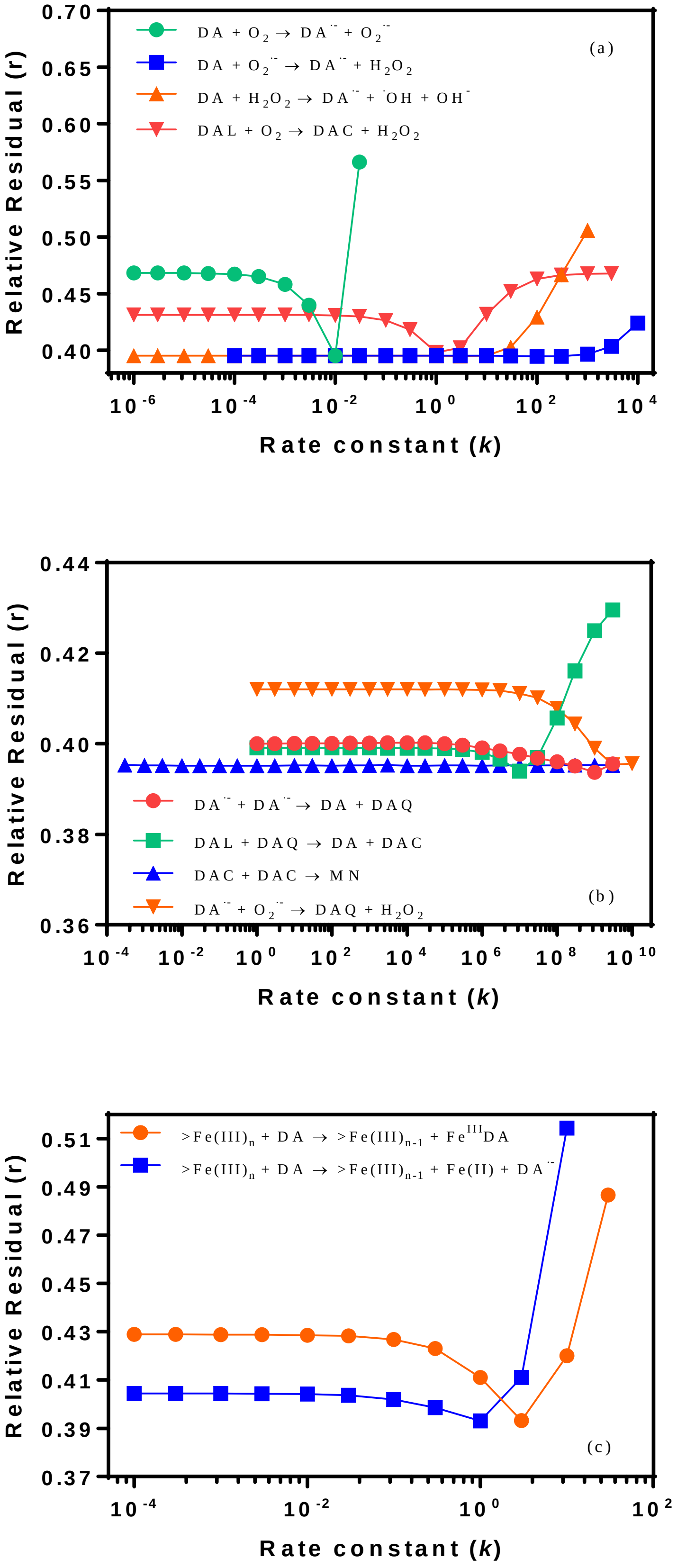

Relative Residual (r)
Rate constant (k)
(a)
DA + O2 → DA˙ + O2˙
DA + O2˙ → DA˙ + H2O2
DA + H2O2 → DA˙ + ˙OH + OH
DAL + O2 → DAC + H2O2
0.70
0.65
0.60
0.55
0.50
0.45
0.40
10 -6
10 -4
10 -2
10 0
10 2
10 4
(b)
DA˙ + DA˙ → DA + DAQ
DAL + DAQ → DA + DAC
DAC + DAC → MN
DA˙ + O2˙ → DAQ + H2O2
0.44
0.42
0.40
0.38
0.36
10 -4
10 -2
10 0
10 2
10 4
10 6
10 8
10 10
(c)
>Fe(III)n + DA → >Fe(III)n-1 + FeIIIDA
>Fe(III)n + DA → >Fe(III)n-1 + Fe(II) + DA˙
0.51
0.49
0.47
0.45
0.43
0.41
0.39
0.37
10 -4
10 -2
10 0
10 2

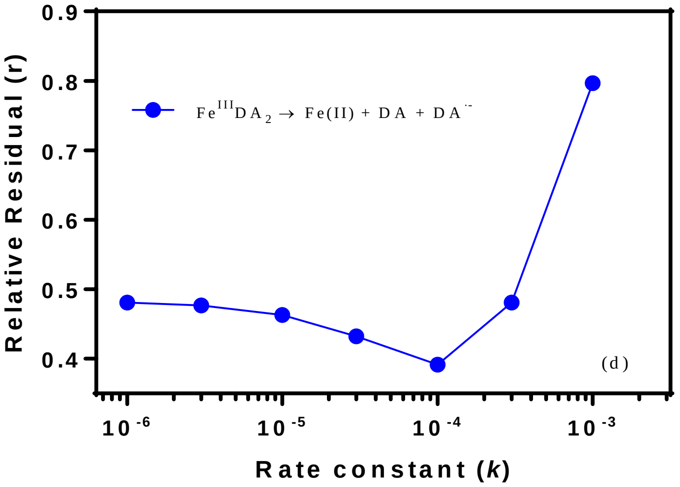
Relative Residual (r)
0.9
0.8
0.7
0.6
0.5
0.4
Fe^III DA2 → Fe(II) + DA + DA·⁻
(d)
10 -6
10 -5
10 -4
10 -3
Rate constant (k)

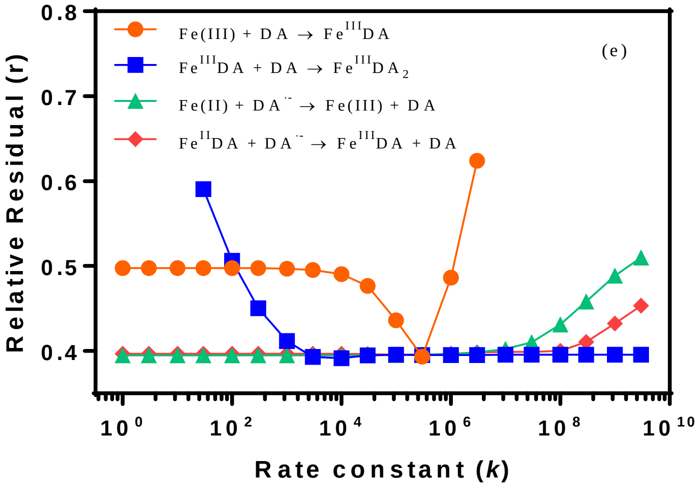
Relative Residual (r)
0.8
0.7
0.6
0.5
0.4
Fe(III) + DA → Fe^III DA
Fe^III DA + DA → Fe^III DA2
Fe(II) + DA·⁻ → Fe(III) + DA
Fe^II DA + DA·⁻ → Fe^III DA + DA
(e)
10 0
10 2
10 4
10 6
10 8
10 10
Rate constant (k)

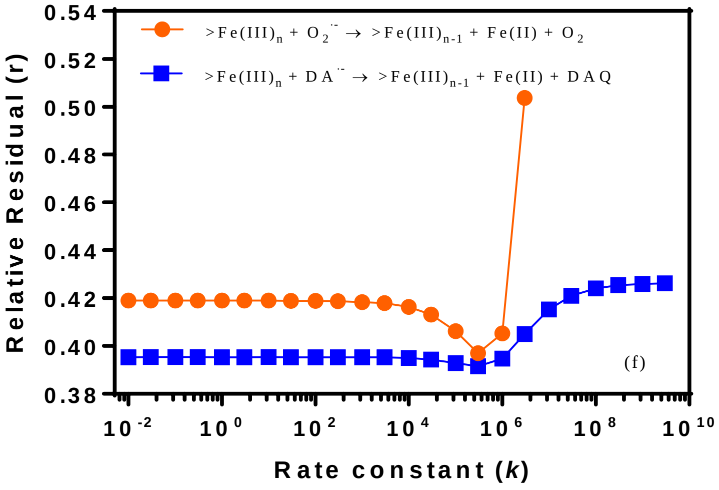
Relative Residual (r)
0.54
0.52
0.50
0.48
0.46
0.44
0.42
0.40
0.38
>Fe(III)n + O2·⁻ → >Fe(III)n-1 + Fe(II) + O2
>Fe(III)n + DA·⁻ → >Fe(III)n-1 + Fe(II) + DAQ
(f)
10 -2
10 0
10 2
10 4
10 6
10 8
10 10
Rate constant (k)

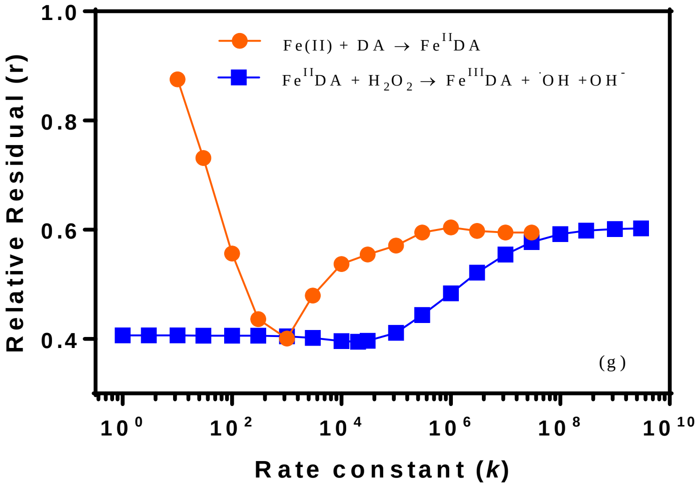
1.0
0.8
0.6
0.4
Relative Residual (r)
Fe(II) + DA → Fe^IIDA
Fe^IIDA + H2O2 → Fe^IIIDA + ·OH +OH⁻
(g)
10⁰
10²
10⁴
10⁶
10⁸
10¹⁰
Rate constant (k)

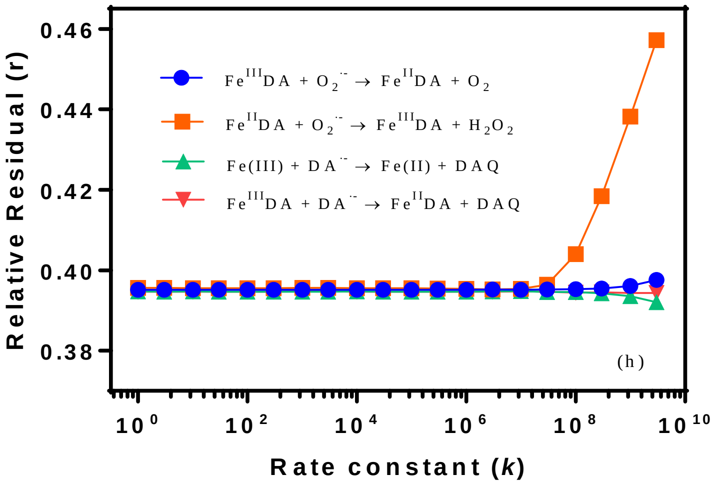
0.46
0.44
0.42
0.40
0.38
Relative Residual (r)
Fe^IIIDA + O2·⁻ → Fe^IIDA + O2
Fe^IIDA + O2·⁻ → Fe^IIIDA + H2O2
Fe(III) + DA·⁻ → Fe(II) + DAQ
Fe^IIIDA + DA·⁻ → Fe^IIDA + DAQ
(h)
10⁰
10²
10⁴
10⁶
10⁸
10¹⁰
Rate constant (k)

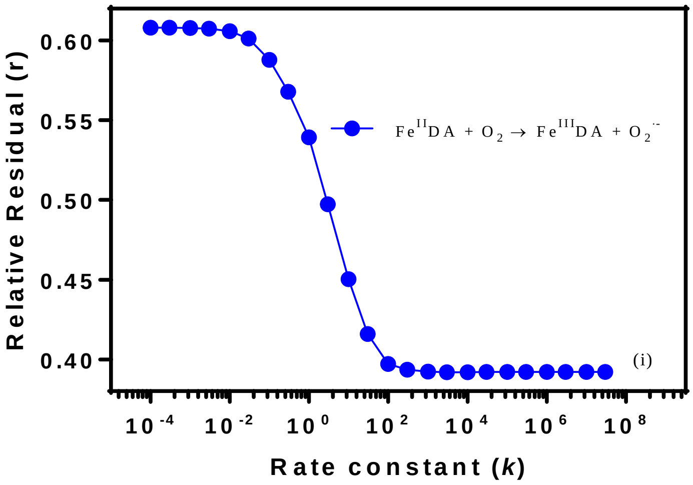
0.60
0.55
0.50
0.45
0.40
Relative Residual (r)
Fe^IIDA + O2 → Fe^IIIDA + O2·⁻
(i)
10⁻⁴
10⁻²
10⁰
10²
10⁴
10⁶
10⁸
Rate constant (k)

Figure S4. Sensitivity analysis for different fitting reaction rate constants (Tables 1 - 3, main text) and some other unimportant reactions.

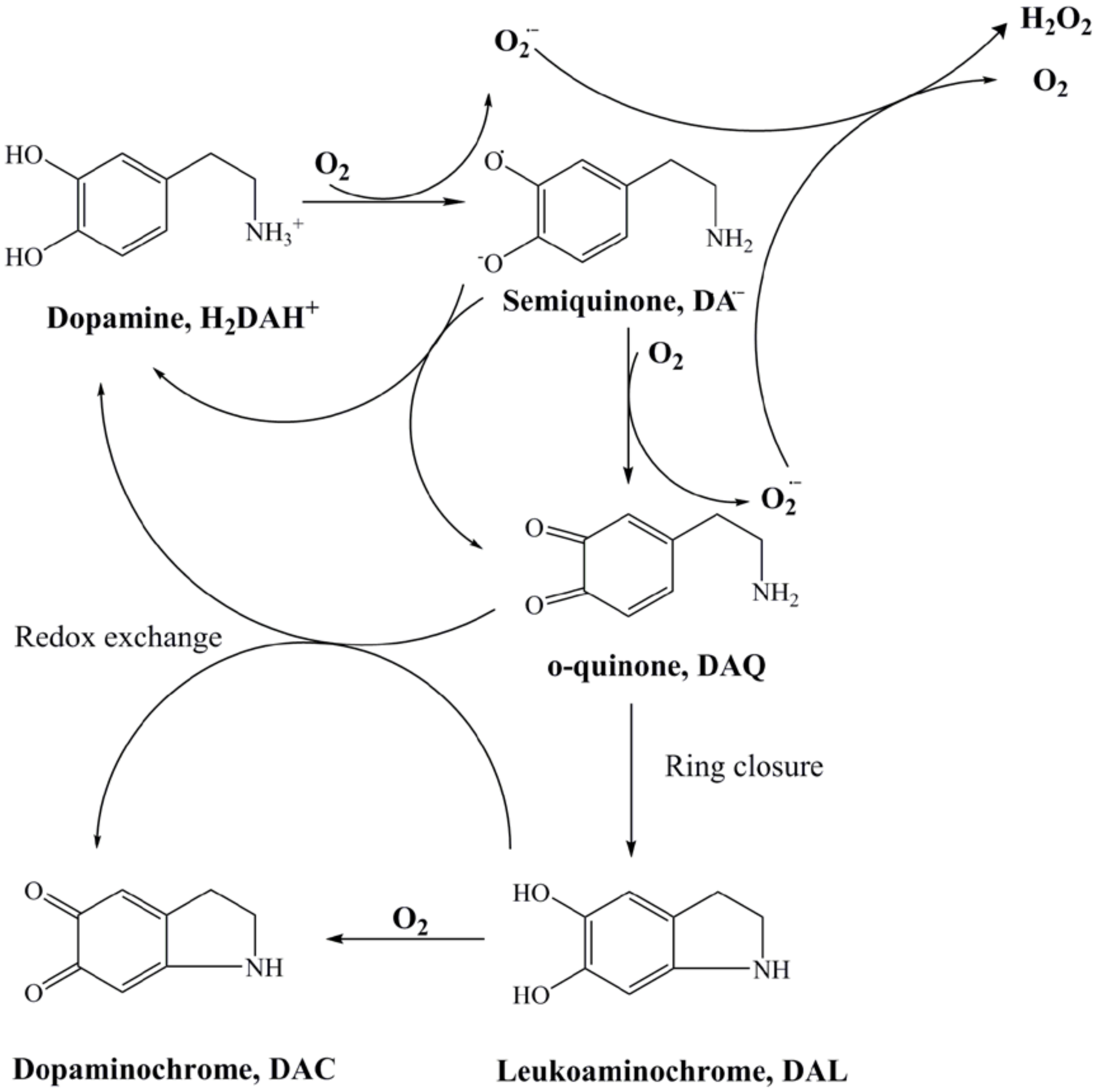

Figure S5. Oxidation pathway of dopamine in the absence of added metals (Pham and Waite 2014)

Text S1

The standard free energy change (ΔG^0) for $Fe^{II}DA$ oxidation to $Fe^{III}DA$ (via one-electron reduction of O_2 to $O_2^{\bullet-}$) is given by

$$\Delta G^0 = -F(E^0_{O_2 \rightarrow O_2^{\bullet-}} - E^0_{Fe^{III}DA \rightarrow Fe^{II}DA})$$

Where $E^0_{O_2 \rightarrow O_2^{\bullet-}} = -0.18$ V (Koppenol *et al.* 2010), *F* is the Faraday constant, $E^0_{Fe^{III}DA \rightarrow Fe^{II}DA}$ is determined using the Nernst equation (Morel and Hering 1993).

$$E^0_{Fe^{III}DA \rightarrow Fe^{II}DA} = E^0_{(Fe^{3+} \rightarrow Fe^{2+})} - 0.059\log_{10}\left(\frac{K_{Fe^{III}DA}}{K_{Fe^{II}DA}}\right)$$

where $E^0_{(Fe^{3+} \rightarrow Fe^{2+})} = 0.77$ V (Schumb *et al.* 1937) is the standard reduction potential of Fe^{3+} and *Ki* is the stability constant of the complex $Fe^{II}DA$ and $Fe^{III}DA$ shown in Table S1. Thus, the calculated redox potential of the complexed iron couple should be $E^0_{Fe^{III}DA \rightarrow Fe^{II}DA} = 0.044$.

References

Avdeef, A., Sofen, S. R., Bregante, T. L. and Raymond, K. N. (1978) Coordination chemistry of microbial iron transport compounds. 9. Stability constants for catechol models of enterobactin. *J Am Chem Soc,* **100,** 5362-5370.

King, D. W. (1998) Role of carbonate speciation on the oxidation rate of Fe (II) in aquatic systems. *Environ Sci Technol,* **32,** 2997-3003.

Koppenol, W. H., Stanbury, D. M. and Bounds, P. L. (2010) Electrode potentials of partially reduced oxygen species, from dioxygen to water. *Free Radical Bio Med,* **49,** 317-322.

Millero, F. J. and Hawke, D. J. (1992) Ionic interactions of divalent metals in natural waters. *Mar Chem,* **40,** 19-48.

Millero, F. J., Yao, W. and Aicher, J. (1995) The speciation of Fe (II) and Fe (III) in natural waters. *Mar Chem,* **50,** 21-39.

Morel, F. M. and Hering, J. G. (1993) *Principles and applications of aquatic chemistry*. Wiley, New York.

Pham, A. N., Rose, A. L., Feitz, A. J. and Waite, T. D. (2006) Kinetics of Fe(III) precipitation in aqueous solutions at pH 6.0–9.5 and 25°C. *Geochim Cosmochim Acta,* **70,** 640-650.

Pham, A. N. and Waite, T. D. (2014) Cu (II)-catalyzed oxidation of dopamine in aqueous solutions: Mechanism and kinetics. *J Inorg Biochem,* **137,** 74-84.

Schecher, W. D. and McAvoy, D. C. (1992) MINEQL+: a software environment for chemical equilibrium modeling. *Computers, Environment and Urban Systems,* **16,** 65-76.

Schumb, W. C., Sherrill, M. S. and Sweetser, S. B. (1937) The measurement of the molal ferric-ferrous electrode potential. *J Am Chem Soc,* **59,** 2360-2365.

Smith, R. M. and Martell, A. E. (1989) *Critical stability constants, Vol. 6: Second Supplement*. Plenum Press, New York, USA.

Yuan, X., Pham, A. N., Xing, G., Rose, A. L. and Waite, T. D. (2012) Effects of pH, chloride, and bicarbonate on Cu(I) oxidation kinetics at circumneutral pH. *Environ Sci Technol,* **46,** 1527-1535.
